# Bioconcentration of Several Series of Cationic Surfactants in Rainbow Trout

**DOI:** 10.1021/acs.est.1c02063

**Published:** 2021-06-16

**Authors:** Amelie Kierkegaard, Marcus Sundbom, Bo Yuan, James M. Armitage, Jon A. Arnot, Steven T. J. Droge, Michael S. McLachlan

**Affiliations:** † Department of Environmental Science, Stockholm University, Stockholm SE-106 91, Sweden; ‡ AES Armitage Environmental Sciences, Incorporated, Ottawa, Ontario K1L 8C3, Canada; § ARC Arnot Research and Consulting, Incorporated, Toronto, Ontario M4M 1W4, Canada; ∥ Department of Physical and Environmental Sciences, University of Toronto Scarborough, Toronto, Ontario MM1C 1A4, Canada; ⊥ Institute for Biodiversity and Ecosystem Dynamics (IBED), University of Amsterdam, Amsterdam 1090 GE, The Netherlands; # Dutch Board for the Authorisation of Plant Protection Products and Biocides (Ctgb), Ede 6717 LL, The Netherlands

**Keywords:** alkylamines, quaternary ammonium, pH dependence, BCF, fish, kinetic model

## Abstract

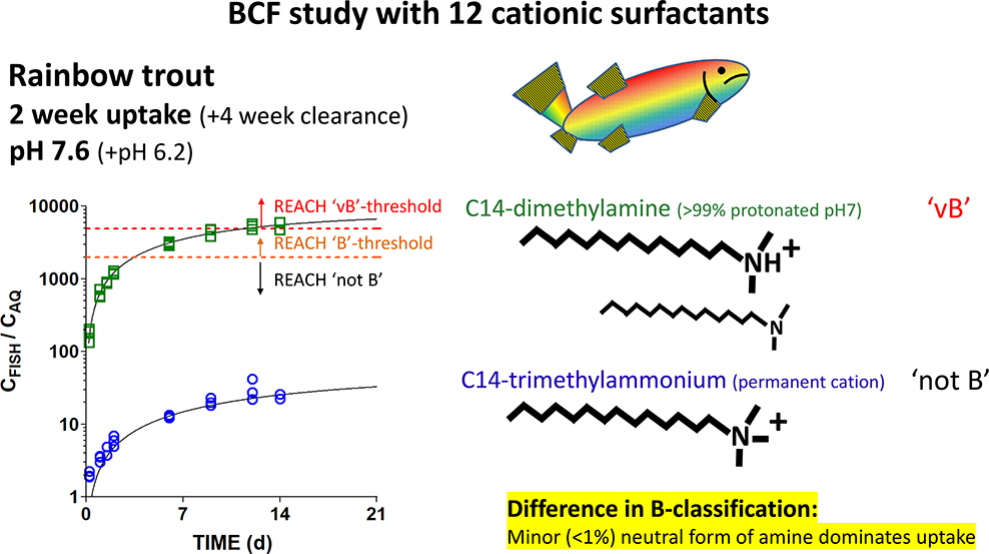

Cationic surfactants have a strong affinity to sorb to phospholipid membranes and thus possess an inherent potential to bioaccumulate, but there are few measurements of bioconcentration in fish. We measured the bioconcentration of 10 alkylamines plus two quaternary ammonium compounds in juvenile rainbow trout at pH 7.6, and repeated the measurements at pH 6.2 for 6 of these surfactants. The BCF of the amines with chain lengths ≤ C_14_ was positively correlated with chain length, increasing ∼0.5 log units per carbon. Their BCF was also pH dependent and approximately proportional to the neutral fraction of the amine in the water. The BCFs of the quaternary ammonium compounds showed no pH dependence and were >2 orders of magnitude less than for amines of the same chain length at pH 7.6. This indicates that systemic uptake of permanently charged cationic surfactants is limited. The behavior of the quaternary ammonium compounds and the two C_16_ amines studied was consistent with previous observations that these surfactants accumulate primarily to the gills and external surfaces of the fish. At pH 7.6 the BCF exceeded 2000 L kg^–1^ for 4 amines with chains ≥ C_13_, showing that bioconcentration can be considerable for some longer chained cationic surfactants.

## Abstract 2

Bioconcentration of cationic surfactants depends on pH and alkyl chain length, with BCFs up to 8200 L kg^−1^ observed.

## Introduction

Cationic surfactants have a wide range of uses, both in consumer goods (e.g., fabric softeners, hair care products, and biocidal ingredients) and industrial processes (e.g., in hydraulic fracturing fluids, pesticide adjuvants).^1−7^ Many are produced in high volumes. In a query of the registration dossiers under the European chemical legislation REACH we identified 29 cationic surfactants with production volumes in excess of 1000 tonnes/year (Table S1 of the Supporting Information, SI).

Bioaccumulation is an important consideration in chemical assessment and regulation.^8^ Cationic surfactants sorb very strongly to phospholipid membranes,^9^ which affords them with a powerful mechanism for bioaccumulation. This provides a particularly strong incentive for bioaccumulation assessment of this class of chemicals. The metric employed for assessing bioaccumulation in many regulations is the bioconcentration factor (BCF) in fish.^8^ The BCF is usually determined according to the OECD 305 protocol, which requires exposure of fish to constant aqueous concentration of the chemical.^10^ However, maintaining constant exposure concentrations has proven difficult for cationic surfactants as many of them possess a strong tendency to sorb to surfaces.^11,12^ As a consequence, there are very few BCF values for surfactants reported in the literature.^13^ A review conducted in 2007 concluded that there were no useful BCF data for cationic surfactants at that time,^14^ and few data have emerged since. We are aware of just two regulatory dossiers that refer to unpublished BCF studies for didecyldimethylammonium chloride and alkyl(C_12–16_)dimethylbenzyl ammonium chloride.^15,16^ Field bioaccumulation factors of long-chain cationic surfactants (C_13_–C_18_) have been reported in the literature, but to a very limited extent.^17^

In the absence of BCF data, other approaches are applied for assessing bioaccumulation. The simplest is to use a partition coefficient as a proxy for the accumulation capacity of the fish. The octanol–water partition coefficient (*K*_OW_) has proven to be a useful proxy for the bioaccumulation potential of neutral organic chemicals, as neutral lipids are the major contributor to fish storage capacity of these chemicals.^18^ However, as has been discussed for anionic surfactants such as perfluorinated acids,^19^ phospholipids are likely to have a much higher storage capacity for cationic surfactants than neutral lipids. The membrane lipid–water distribution ratio (*D*_MLW_) has been proposed as a much more useful bioaccumulation potential proxy than *K*_OW_ for cationic surfactants.^9^

Bioconcentration does not just depend on the fish’s storage capacity; it is also determined by the ability of the fish to take up the chemical and to biotransform it.^20,21^ Transport across membranes is typically much slower for charged organic ions than for neutral chemicals,^22−25^ and cations are often rapidly metabolized in vivo.^26^ Therefore, an evaluation of bioaccumulation of cationic surfactants based only on a proxy for fish storage capacity risks considerably overestimating the BCF. To overcome this limitation, more complex approaches for assessing bioconcentration are being developed that combine in silico or in vitro estimates of biotransformation^27,28^ with mechanistic models of bioaccumulation^29,30^ to predict more refined BCF values. In order for these methods to gain acceptance and reach their full potential, in vivo BCF data are required to evaluate their performance.

To address this need, we measured the BCFs of 12 cationic surfactants in rainbow trout (*Oncorhynchus mykiss*) based on the OECD 305 protocol. Since this was a research investigation and not a regulatory study, we modified the OECD protocol to suit the research questions, borrowing some elements of the BETTER BCF protocol.^31^ Alkylamines, an important class of cationic surfactants (see Table S1), were chosen as the test chemicals. The log *D*_MLW_ of alkylamines increases proportionally with alkyl chain length.^9^ Hence a range of chain lengths was included, starting with C_9_ and extending to C_16_, the longest chain length for which a BCF determination according to OECD 305 was judged possible. By studying the chain length dependence, we sought to develop an ability to extrapolate BCF models to even longer alkyl chained surfactants for which BCF measurements are not possible. Primary, secondary, and tertiary alkylamines were included, as the headgroup can influence biotransformation. Finally, we tested two quaternary ammonium compounds (QACs), permanently charged analogues of the tertiary amines, to provide insight into differences in the bioaccumulation of the charged and neutral forms. BCF experiments were conducted with 2 mixtures of 6 surfactants each. For one of the mixtures the experiment was conducted at two pHs, as pH influences cationic surfactant dissociation in the external environment and thus, potentially, bioaccumulation. In addition to determining the BCF, we also studied uptake and elimination kinetics and applied a mass balance model to obtain more mechanistic insight and explore whether elimination behavior is consistent with current understanding of partitioning into fish tissue. An earlier study about tissue distribution of cationic surfactants indicated that sorption to skin/mucus, sorption to gills, and systemic absorption contribute significantly to body burden.^32^ However, because it is difficult to physically separate residues originating from these pathways, whole fish were analyzed.

## Methods

### Test Chemicals and Reagents

The test chemicals consisted of ten alkylamines (including four primary amines (abbreviated P), two secondary amines (S) and four tertiary amines (T)) and two QACs (Q) (see Table 1 for the names and abbreviations, Table S2 in the SI for the suppliers, and Table S3 for properties). Two mixtures of six test chemicals were used, whereby the chemicals were grouped such that each chemical in a mixture had a different alkyl chain length. This ensured that one test chemical could not be formed by demethylation of the amine group of another test chemical. The same test chemical mixtures were used in the earlier study of tissue distribution.^32^

**Table 1:** Test Chemicals Used in the Three Experiments with Abbreviations and Concentrations in Aquarium Water

		concentration in aquarium		
abbreviation	molecular formula (p*K*_a_used)	target	measured (μg L^–1^)^a^	% of target
MIX1				
P9	C_9_NH_2_ (10.6)	100	104 (6)	104
T10	C_10_N(CH_3_)_2_ (10.0)	25	18.9 (5)	76
P12	C_12_NH_2_ (10.6)	25	23.8 (6)	95
T13	C_13_N(CH_3_)_2_ (10.0)	10	7.4 (6)	74
Q14	C_14_N(CH_3_)_3_^+^ (−)	25	25.1 (4)	100
P16	C_16_NH_2_ (10.6)	2.5	1.53 (6)	61
MIX1pH				
P9	C_9_NH_2_ (10.6)	100	112 (4)	112
T10	C_10_N(CH_3_)_2_ (10.0)	25	22.1 (5)	88
P12	C_12_NH_2_ (10.6)	25	28.7 (4)	115
T13	C_13_N(CH_3_)_2_ (10.0)	10	7.9 (5)	79
Q14	C_14_N(CH_3_)_3_^+^ (−)	25	26.0 (4)	104
P16	C_16_NH_2_ (10.6)	2.5	1.56 (7)	62
MIX2				
T9	C_13_N(CH_3_)_2_ (10.0)	50	50 (3)	100
Q10	C_10_N(CH_3_)_3_^+^ (−)	50	55 (2)	110
S12	C_12_N(CH_3_)H (10.8)	25	22.3 (4)	89
P13	C_13_NH_2_ (10.6)	10	6.5 (4)	65
T14	C_14_N(CH_3_)_2_ (10.0)	2.5	1.71 (6)	69
S16	C_16_N(CH_3_)H (10.8)	2.5	1.66 (9)	67

a Mean (relative standard deviation in %).

The quality and supplier of the solvents used are listed in Table S4. Analytical standards were prepared in methanol and stored in glass. Polypropylene vials were employed for storing all extracts and solutions.

### Fish Exposure and Sampling

Juvenile rainbow trout were purchased from EM lax in Västervik, Sweden, and held in the aquaria facility for one month prior to starting the experiment on August 28, 2018. Ethical approval for the experiments was obtained from Stockholm’s djurförsöksetiska nämnd (decision 9967–2017). The experiment was conducted in six 350 L fiberglass aquaria with a water renewal rate of 1.5 L min^–1^ corresponding to a turnover time of ∼3.3 h. The water temperature and the air temperature in the aquaria room were 10 °C. The lighting was dim and programmed on a 12 h light/12 h dark cycle.

Each aquarium was equipped with external circulating pumps (Eheim model 2273 Prof 4) containing a filter of polyester wool wadding. Aquarium water was pumped at 800–1200 L h^–1^, and the water discharge hose was placed on the screen covering the aquarium, so that the resulting bubble entrainment provided for aeration. Each pump was equipped with a prefilter housing (Eheim) that was filled with the same polyester wool. The prefilter wadding was exchanged daily, while the wadding in the pump filter was replaced twice over the duration of the experiment. To further reduce the levels of organic material in the aquaria, the fish feces were siphoned off of the bottom of the aquaria each day, 1 h after the fish were fed.

The fish were fed once daily using fish pellets supplied by the fish farm at ∼1% of their body weight per day. Of the ∼375 fish used in the experiment, 2 died as a result of jumping out of the aquaria, 4 had body deformities (swollen abdomen; these fish were not analyzed), and 5 had eye injuries.

Three BCF experiments were conducted, one each for MIX1 and MIX2 at pH ∼7.6 (denoted MIX1 and MIX2 in the following), and one for MIX1 at pH ∼6.2 (MIX1pH). Each experiment consisted of a 14 d exposure phase and a 28 d depuration phase. There were also two control aquaria where unexposed fish were kept, one at pH 7.6 and one at pH 6.2. For the pH 6.2 aquaria, a 20% v:v formic acid solution was pumped into a premixing basin where it was mixed with the inflowing water before entering the aquarium. pH sensors monitored pH in the premixing basin and the aquarium. The pH for the MIX1 and MIX2 experiments was governed by the pH of the water supply. Water samples were collected at 8 time points during each experiment for the determination of pH and alkalinity. Water samples were collected daily during the exposure phase for determination of total organic carbon (TOC), which was measured with NDIR detection after acidification, purging and high-temperature catalytic combustion (Shimadzu TOC-L).

For the exposure phase, a methanol solution of the test chemical mixture was infused continuously at 4.0 μL min^–1^ into the water inflow of the aquarium using a syringe pump. The target concentrations of the chemicals in water ranged from 2.5 to 100 μg L^–1^ (Table 1). They were selected to be as low as possible to minimize the risk of toxic effects while still being high enough to enable collection of high-quality data, building on the results of the tissue distribution experiment.^32^ The syringe pump was started in an aquarium containing no fish. After 16 h to allow the concentrations to stabilize, ∼80 rainbow trout were transferred to the aquarium. Over the following 14 d water samples were collected and fish were sacrificed according to the schedule in Table S5. The remaining fish were then transferred to a second aquarium that received water without chemical addition. Over the following 28 d further water samples were collected and fish were sacrificed (Table S5).

The water samples were collected ∼30 min after the daily removal of feces from the aquarium. Triplicate samples were taken at each time point. An autopipette with a polypropylene tip was pumped 5 times with aquarium water and then 600 μL of aquarium water was sampled and transferred to a 1.5 mL polypropylene vial containing 900 μL of methanol and isotope labeled standards of Q10, Q14, and P16. 60 μL of the water/methanol mixture was then analyzed using LC–MS/MS as described below for fish, modified to accommodate a 100 μL injection loop.

At each fish sampling, 4–6 fish were sacrificed. Fish were also sampled periodically from the control aquaria. Following stunning and severance of the spinal cord, the fish were placed in a polyethylene bag, weighed, and immediately frozen at −20 °C. The median fish weight at sacrifice was 10.94 g, with a 25^th^ and 75^th^ percentile of 8.43 and 13.58 g, respectively.

### Sample Analysis

Three fish from each time point were prioritized for analysis based on proximity to the median fish weight. Each fish was semithawed, cut into 3–4 pieces, and homogenized with an Ultra-Turrax device. 150 mg of the homogenate was transferred to a preweighed 13 mL polypropylene tube, centrifuged to a pellet, and weighed. 50 μL of methanol was added and the prepared aliquot was frozen. For extraction internal standard solution (isotope labeled standards of Q10 (D_21_), Q14 (D_29_), and P16 (D_33_)), 3 mL of methanol and 3 steel balls (3.2 mm diameter) were added to the prepared aliquot. This mixture was mixed in a bullet blender for 1 min, and then placed in an ultrasound bath at 50 °C for 60 min. Following centrifugation, the supernatant was decanted and the extraction was repeated. The extracts were combined and a portion corresponding to 25–50 mg homogenate was cleaned up on a weak cationic exchange SPE column (WCX, 60 mg) as described elsewhere.^32^

For instrumental analysis, 5 μL of purified fish extract (methanol) or 60 μL of the water/methanol mixture (water samples) was separated on an ACQUITY UPLC BEH C18 column, analyzed on a Waters Xevo TQS triple quadrupole mass spectrometer, and quantified against the internal standards as described elsewhere.^32^Text S1, Tables S6–S11, and Figure S1 document different quality aspects of the analyses of test chemicals in water and fish.

### Estimation of BCF

A one compartment BCF model with first order kinetics was fit to the measured concentrations in fish, using the measured concentrations in water as input. More information on the fitting procedure is given in Text S2. The BCF (L kg^–1^) was calculated as the quotient of the uptake rate constant *k*_U_ and the elimination rate constant *k*_T_ (except for Q10, see below).

### Mechanistic Model of Amine Bioconcentration

To gain more mechanistic insight into the results, we formulated a simple one-box mass balance model of chemical bioconcentration in fish that considered diffusive uptake and elimination across the gills as well as biotransformation.^33^ The flow of water across the gills and the flow of blood through the gills were modeled as perfusion limited resistances that constrain gill uptake and elimination. For further resistances to gill exchange posed by cell membranes and cytosol, we did not identify sufficient information to construct a well-constrained model. Consequently, these resistances are unknowns. Ion trapping effects caused by differences in pH between water at the gill surface and blood in the gills are considered, drawing on previous work on the uptake and elimination of ionizable organic chemicals across the gills of rainbow trout.^22,23^ Partitioning of the chemical from the dissolved phase into blood and into whole fish was calculated as the product of the membrane lipid–water distribution ratio (*D*_MLW,T_, including partitioning of both the neutral and the charged forms) and the fraction of membrane lipid in blood and whole fish. A detailed description of the model is provided in the SI (Text S3).

## Results and Discussion

### Water Chemistry

The pH in the MIX1 and MIX2 experiments increased from 7.6 to 7.9 during the experiment, averaging 7.62 during the exposure phase and 7.80 during the elimination phase (Table S12). The pH in the MIX1pH experiment increased from 6.1 and 6.4 during the exposure phase, averaging 6.22, and varied around 6.3 during the elimination phase (Figure S2). The alkalinity averaged 1.282 mmol L^–1^ (Table S12). The TOC content of the aquarium water in the MIX1 and MIX2 experiments was 5.47 ± 0.33 mg C L^–1^ (Table S13).

### Concentrations in Water

There was good agreement between the measured concentrations and target concentration of the test chemicals in water during the exposure phase (Table 1). For the C_9_–C_12_ alkylamines (exception T10) and the QACs the average measured concentration was 88–115% of the target concentration. Values as low as 61% were measured for longer chained alkylamines. This was presumably due to greater sorption losses to surfaces in the aquarium system, particularly surfaces that were regularly renewed such as the polyester wool wadding in the filters and the particulate organic matter that collected therein. Bioconcentration was assessed based on the measured concentrations in water.

The test chemical concentrations were quite stable during the exposure phase (Table 1). The relative standard deviation (RSD) of the average concentration measured at the 20 sampling time points ranged from 2% to 9%. The highest RSDs were measured for two of the most strongly sorbing compounds: S16 (9%) and P16 (7% for the MIX1pH experiment). For all other test chemicals, the RSD was ≤6%. The concentration of all test chemicals in water was markedly lower during the elimination phase than during the exposure phase (Tables S14–S16).

### Concentrations in Fish

The concentrations in the control fish were in the same range as the blanks. The LOQ of the method was calculated as 10× standard deviation of the blank/control fish. All test chemicals were above the LOQ in the fish throughout the exposure phase and at least the first 3 time points during the elimination phase, with the following exceptions: P9 during MIX1, Q10 during MIX2, and P9 and T10 during MIX1pH (Tables S17–S22). If one sample at a given time point was at or below the LOQ, then all data for that time point were disregarded.

The variability in concentrations between fish at a given time point ranged from 0.09 to 0.61 (mean RSD, Table S23). This was considerably in excess of the repeatability of the analytical method, indicating that there were marked individual differences in accumulation between fish. The variability varied greatly between test chemicals, with the mean RSD of the primary amines (45%) much higher than for the secondary amines (13%), tertiary amines (12%), or Q14 (15%). Q10 had a higher mean RSD (34%), but this can be attributed at least in part to the proximity of the concentrations to the LOQ. Possible causes of the individual variability include differences in ventilation rate (which would influence the rate of uptake and possibly elimination) and in biotransformation. Individual variation in respiration is unlikely to explain the high variability of the primary amines, both because the magnitude of the variation is much greater than the plausible variation in ventilation rate and because ventilation would be expected to affect most test chemicals in the same mixture to a similar extent. For individual variation in biotransformation to explain the variability, other elimination mechanisms such as ventilation would have to be negligible. Another possible explanation is interindividual differences in the skin mucus burden. Our previous study of tissue distribution indicated that 4–30% of the fish’s body burden of the test chemicals was associated with the skin mucus, with the primary amines showing the largest fractions.^32^ However, the differences in the mucus-associated fraction between primary and tertiary amines were much less than the differences in interindividual variability in concentration between these substance groups, which would speak against this explanation.

Semiquantitative screening of possible demethylation metabolites was conducted in the fish collected at the last time point of the exposure phase. Secondary amines were observed as metabolites of all 4 of the tertiary amines, whereby the concentration relative to the parent compound decreased with increasing chain length (Table S24). For T9 and T13, trace levels of the corresponding primary amines (P9 and P13, respectively) were also observed, but at levels at least an order of magnitude lower than S9 and S13. P12, a metabolite of S12, was also observed, but P16 was not detected in the fish exposed to S16. Demethylation of T10 to form S10 was reported in an in vitro S9 assay, and it was proposed that this is an important elimination mechanism for tertiary amines in fish.^27^ There is in vitro evidence that demethylation of secondary alkylamines also occurs, which will be reported elsewhere.^37^ Our observations show that demethylation also occurs in vivo.

### Bioconcentration Factors (BCFs)

Semilogarithmic plots of concentration in fish during the elimination phase of the pH 7.6 experiments versus time yielded linear relationships for most test chemicals (Figures S3–S5), indicating that elimination kinetics were first order and that a one compartment model could be applied to estimate the BCF. T10 was a notable exception, showing a pronounced decrease in elimination rate after 48 h at concentrations that were still well above the LOQ (Figure S3). However, the decrease in water concentration over this period (∼100×) was not much greater than the decrease in fish concentration (∼40×), so it is possible that the slower elimination after 48 h was due to a smaller concentration gradient between fish and water. Only the data from the first 48 h were used for estimating the kinetic rate constants and BCF of T10.

There was generally a good fit between the modeled and observed concentrations in fish (Figures S6–S8), whereby the high variability in the observed concentrations of the primary amines resulted in higher uncertainty in the fitted model parameters for these substances. Many of the observed concentrations of Q10 were below the LOD, so it was not possible to fit the kinetic model to the data to derive rate constants. Instead, an upper bound of the BCF for Q10 was estimated using the average of the concentrations above the LOQ during the exposure phase of the experiment.

The BCF ranged from <0.1 for Q10 to 8200 L kg^–1^ for S16 (Table 2). Four of the test chemicals (T13, T14, P16, and S16) had BCFs greater than 2000 L kg ww^–1^, the threshold for classification as a bioaccumulative substance (“B”) in the REACH regulation. Three of the chemicals (T14, P16, and S16) had BCFs greater than 5000 L kg ww^–1^, the threshold for a very bioaccumulative substance (“vB”). This indicates that bioaccumulation of cationic surfactants can be relevant in a regulatory context and underscores the need for better understanding of the underlying processes.

**Table 2:** Measured Uptake Rate Constant *k*_U_, Overall Elimination Rate Constant *k*_T_ and BCF, and Comparison of *k*_T_ with the Gill Elimination Rate Constant *k*_2_

	uptake rate constant (*k*_U_, L kg^–1^ h^–1^)	measured total elimination rate constant (k_T_, h^–1^)	BCF (*k*_U_/k_T_, L kg^–1^)	predicted gill elimination rate constant eq 1 (*k*_2_, h^–1^)	*k*_2_/k_T_
MIX1					
P9	nq^a^	nq	2.2		
T10	9.9	0.074	134	0.37	5.0
P12	2.0	0.0081	250	0.0009	0.11
T13	15.9	0.0049	3200	0.010	1.9
Q14	0.104	0.0020	51		
P16	15.3	0.0025	6100	0.00003	0.01
MIX1pH					
P9	nq	nq	0.1^b^		
T10	1.02	0.129	7.9	0.53	4.1
P12	0.36	0.051	7.1	0.0022	0.04
T13	4.7	0.0139	340	0.039	2.8
Q14	0.121	0.0022	55		
P16	7.8	0.0026	3000	0.00023	0.09
MIX2					
T9	2.0	0.059	35	0.27	4.6
Q10	nq	nq	<0.2^c^		
S12	5.5	0.0066	840	0.004	0.6
P13	4.8	0.0034	1390	0.0006	0.17
T14	27	0.0034	8100	0.004	1.2
S16	22	0.0027	8200	0.00007	0.03

a nq = not quantifiable

b Estimated from upper range of concentrations in fish, all of which were below the LOQ.

c Estimated from concentrations in fish during what is believed to be the plateau phase, which were just above the LOQ.

**Figure 1:**
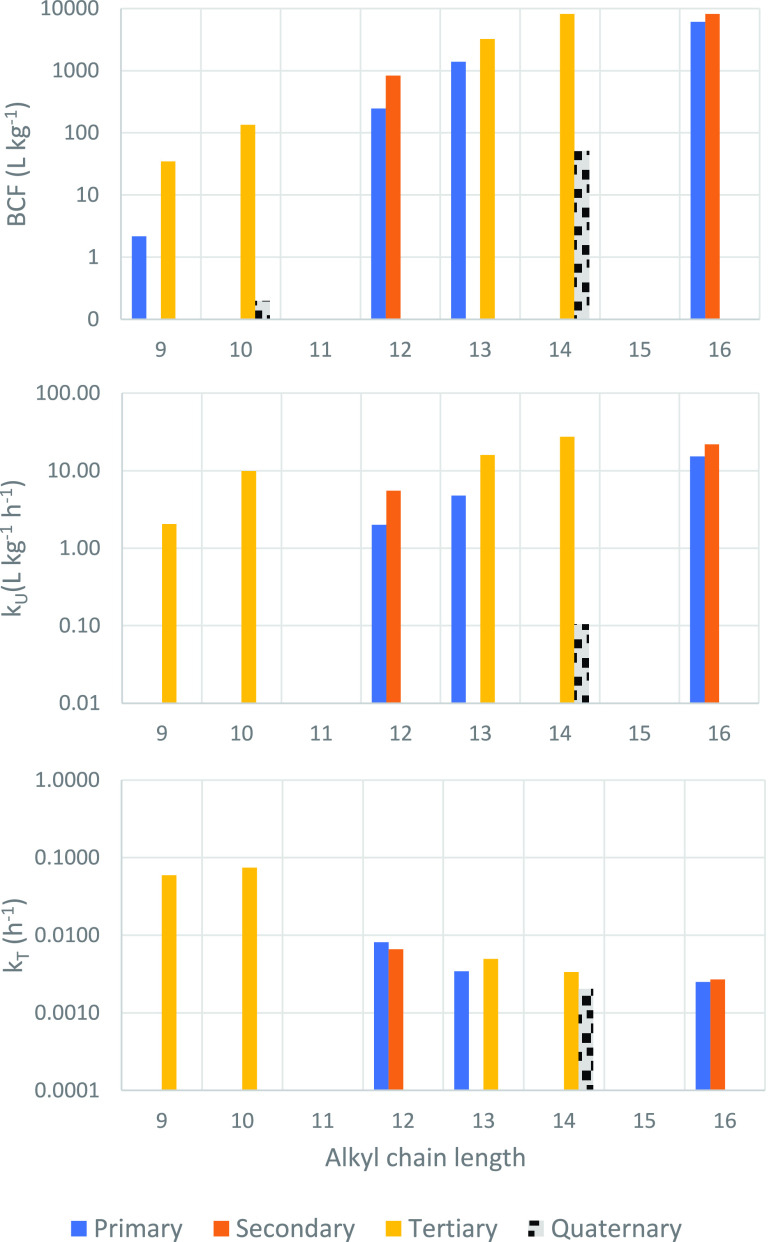
Bioconcentration parameters of all test chemicals at pH 7.6: BCF (upper panel, L kg^–1^); *k*_U_ (middle panel, L kg^–1^ h^–1^); *k*_T_ (lower panel, h^–1^).

A positive correlation between BCF and the alkyl chain length was observed for the amines with ≤C_14_, whereby BCF increased ∼0.5 log units for each additional carbon in the chain (Figure 1, upper panel). The substitution of the amine group had a smaller effect on BCF, but there was a consistent trend of primary amines being less bioaccumulative than secondary and tertiary analogues. The positive correlation between BCF and alkyl chain length did not apply to the longest chain length chemicals, P16 and S16. It is possible that the dissolved (bioavailable) concentration of the C_16_ cationic surfactants was less than the measured value because of sorption to organic matter in the water. However, an estimate of the fraction sorbed using the measured TOC in the aquarium and literature estimates of the organic carbon–water distribution ratio suggested that this effect was small (see Text S4).

The BCF was markedly lower for the QACs compared to tertiary amines with the same chain length: a factor >670 for Q10 compared to T10, and a factor 160 for Q14 compared to T14 (Figure 1, upper panel). The prominent structural difference between the QACs and the analogous tertiary amines is that the former are permanently charged, while the latter exist in both a charged (protonated) and a neutral form. With an estimated p*K*_a_ of 10, the average neutral fraction of the tertiary amines in the water at the fish gill surface during the pH 7.6 experiments was estimated to be 0.002 (see Text S5). Hence, the presence of a neutral fraction of 0.2% resulted in BCFs that are more than 2 orders of magnitude higher than for a similar compound with no neutral fraction.

### Uptake Rate Constant

More insight into the variability in BCF can be obtained by studying the uptake and elimination kinetics. The variability in the uptake rate constant k_U_ of the amines was small in comparison with the variability observed for BCF (Figure 1, middle panel). With the exception of T9 and P12, *k*_U_ ranged from 5 to 27 L kg^–1^ h^–1^, or 120 to 650 L kg^–1^ d^–1^ (Table 2). In BCF experiments in the same aquarium facility with somewhat larger rainbow trout we measured a median *k*_U_ of 266 L kg^–1^ d^–1^ for 10 neutral chemicals.^31^ This lies well within the range measured for the cationic surfactants. Later in the paper we discuss the factors affecting *k*_U_ from a mechanistic perspective.

The *k*_U_ of T14 was a factor of 260 higher than for Q14, a largely analogous molecule except that it is permanently charged. This is evidence that only the neutral fraction of these cationic surfactants is available for uptake across the gills at appreciable rates. Given that the neutral fraction of T14 was approximately 0.002 at the gill surface (see above and Text S5), the factor 260 higher *k*_U_ for T14 is consistent with previous literature demonstrating that membrane permeabilities of charged organic ions are greatly reduced compared to analogous neutral molecules.^34^

### Elimination Rate Constant

For the amines, the elimination rate constant k_T_ varied less than the uptake rate constant (Figure 1, lower panel; Table 2). The C_12_–C_16_ amines had quite similar *k*_T_ (within a factor 3.2), whereby there was a consistent trend within this group of decreasing *k*_T_ with increasing chain length. The *k*_T_ values for the shorter chained amines T9 and T10 were about an order of magnitude larger (faster).

Elimination from internal tissues can occur via respiration (transport across the gills), biotransformation, and fecal egestion, while chemical sorbed to external fish surfaces (skin/mucus and gills) can desorb. Growth dilution can also lead to apparent elimination. The *k*_T_ values (≥5% per day) were considerably larger than could be explained by fish growth (0.57 ± 0.13% per day). We are not aware of measurements of fecal egestion of cationic surfactants. We believe that either respiration or biotransformation is likely to have controlled elimination of the amines from internal tissue.

The quaternary ammonium compound Q14 had a *k*_T_ that was similar to that of the longest chained amines (P16 and S16). In the tissue distribution study Q14 was predominantly associated with the gills and skin/mucus.^32^ Therefore, its elimination is probably dominated by desorption from these tissues.

### Influence of pH

**Figure 2:**
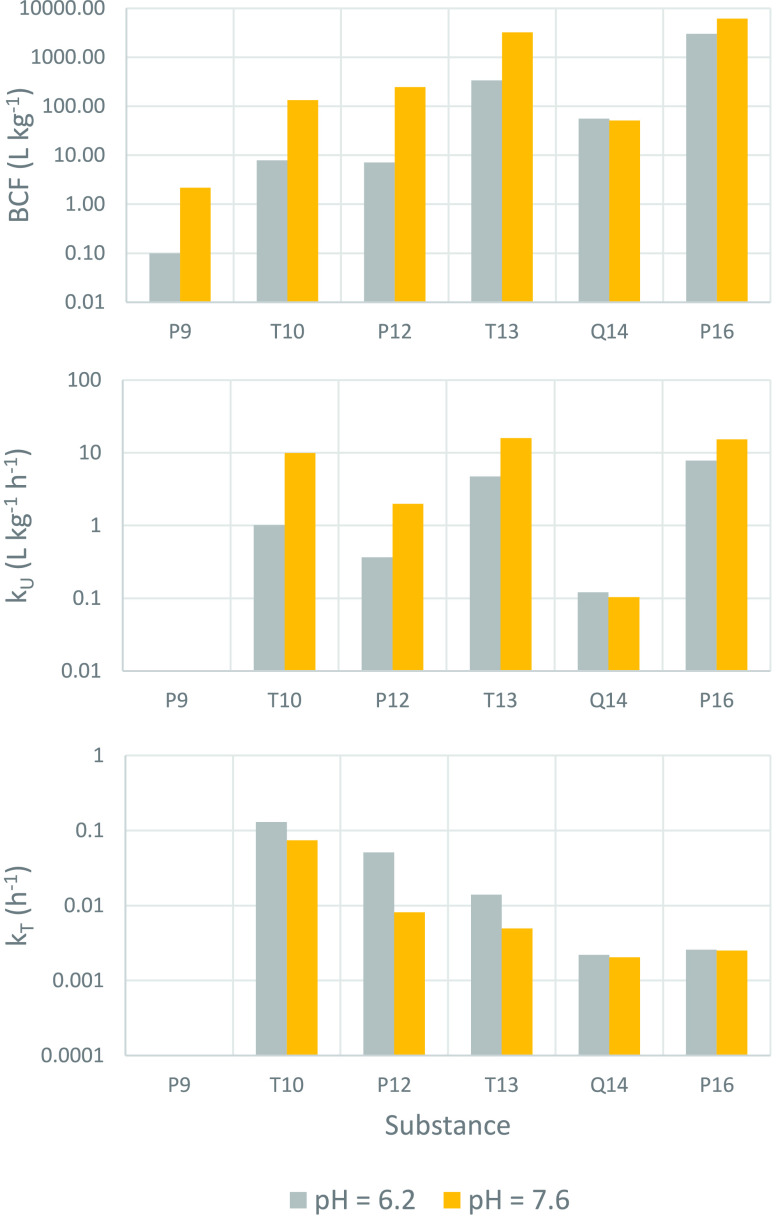
Comparison of bioconcentration parameters at pH 6.2 and at pH 7.6: BCF (upper panel); *k*_U_ (middle panel); and *k*_T_ (lower panel). The BCF for P9 at pH 6.2 is an upper estimate based on concentrations in fish that were below the LOQ.

The BCF was measured at pH 6.2 and pH 7.6 for the six test chemicals in MIX1. The BCF was lower at the lower pH for all of the amines (Figure 2, Table 2). The difference ranged from a factor of 2 for P16 to a factor of 34 for P12. A lower BCF at lower external pH is consistent with predominantly the neutral form of the amine being transported across the gills. In the pH 6.2 experiment the estimated neutral fraction of the amines at the gill surface was 14× smaller than in the pH 7.6 experiment (see Text S5), which corresponded to BCFs that were lower by a factor of 22, 17, 34, and 9.5 for P9, T10, P12, and T13, respectively. For Q14, however, there was no influence of pH on BCF. This result is expected since the QACs are permanently charged and hence there is no neutral fraction that varies with pH.

At pH 6.2, *k*_U_ for the amines was between 2 (P16) and 10 (T10) times lower than at pH 7.6, while it was the same for the permanently charged Q14 (Figure 2). These observations are consistent with limited transport of the charged form of these molecules across the gills. The fact that the difference in *k*_U_ for the amines was less than the difference in the neutral fraction at the gill surface (14×) can be explained by the uptake at pH 7.6 being limited by water flow through the gills, not transport across the gill membranes (see below).

pH had a marked influence on *k*_T_ of P12 and T13, but not on that of the other test chemicals. *k*_T_ for P12 and T13 was 6 and 3 times greater, respectively, at pH 6.2 than at pH 7.6. We discuss this observation below.

### Mechanistic Assessment of Alkylamine Uptake

To gain more mechanistic insight, the results were compared with predictions from the mechanistic model described in the Methods section and SI (Text S3). As a consequence of the findings above, diffusive transport of the cationic form of the alkylamine across the gills was assumed to be negligible. Still, the *D*_MLW_ for the protonated form of alkylamines is most likely the dominant partition process that determines the equilibrium distribution between organic tissue phases and freely dissolved internal concentrations. In contrast to hydrophobic neutral chemicals, the permeation rate across a cell membrane and sorption affinity to the cell membrane are largely independent properties for organic cations.

**Figure 3:**
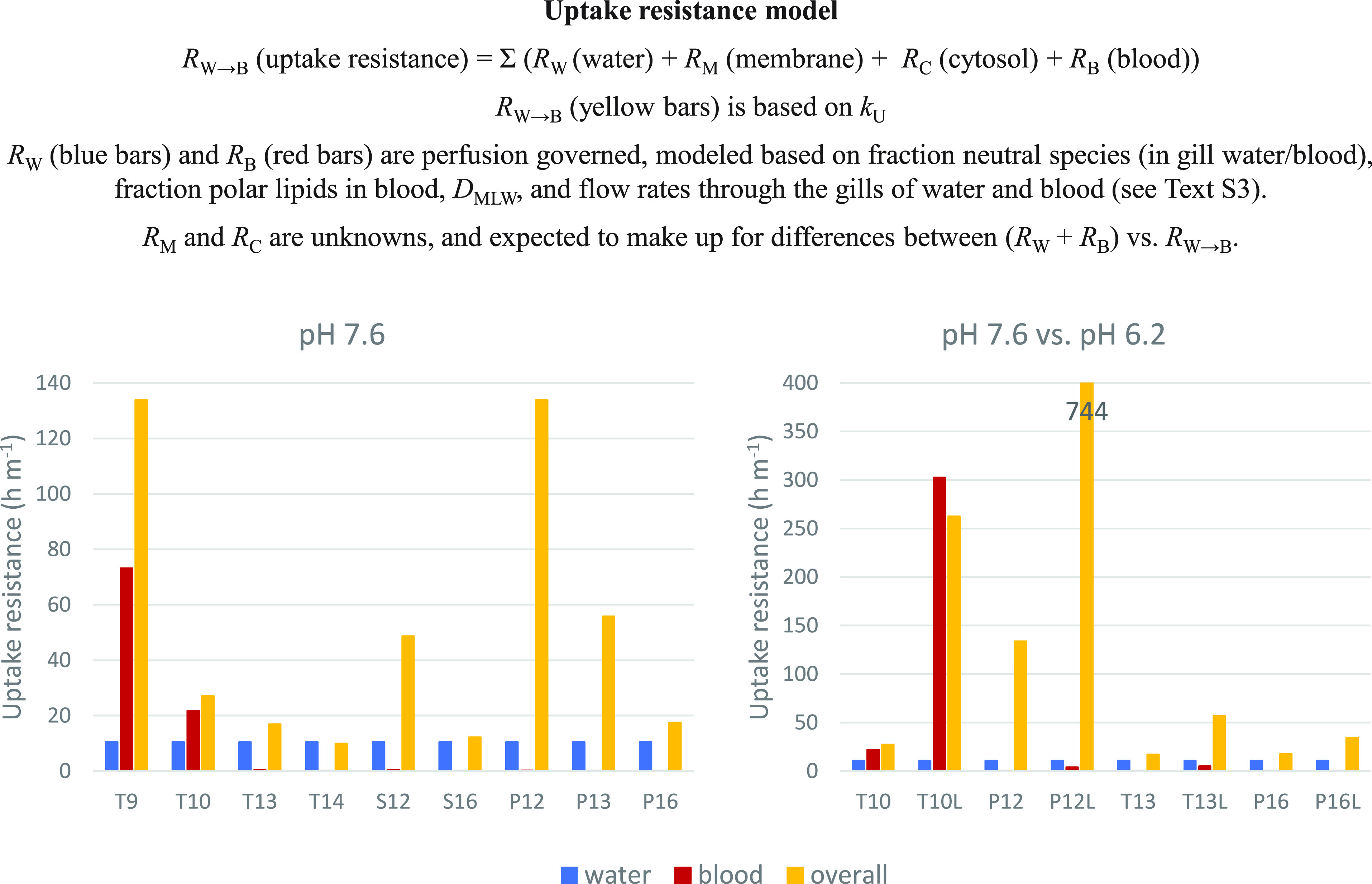
Comparison of the measured overall resistance for uptake of alkylamines with modeled water perfusion and blood perfusion resistances (T# are tertiary amines with # representing alkyl chain length, S# are secondary amines, and P# are primary amines; L indicates lower pH result).

The contribution of the water perfusion and blood perfusion resistances were compared with the overall resistance for transport across the gills from water to blood (*R*_W→B_), which was calculated from the measured *k*_U_ according to equation S4.3. The results for the experiment at pH 7.6 (Figure 3, left panel) show that the blood resistance and the overall resistance were comparable for T9 and T10. This indicates that the uptake rate of T9 and T10 is constrained by the rate of blood delivery to the gills (i.e., cardiac output). For all of the other chemicals the blood resistance is insignificant compared to the overall resistance. The other chemicals have higher *D*_MLW_ and sorb to membrane lipids in the blood, hence enhancing the blood’s capacity to transport the chemicals out of the gills.

The modeled water resistance agrees well with the measured resistance for T14 and S16. This indicates that the uptake of these chemicals is controlled by the amount of water being transported through the gills (i.e., the gill ventilation rate). This water resistance also exceeds 50% of the measured resistance for T13 and P16, indicating that the gill ventilation rate is a significant factor limiting the uptake of these substances.

Neither the blood nor the water resistances constrain the uptake of S12, P12, and P13. This indicates that other resistances, such as those posed by membranes or cytosol in the gill epithelium, dominate for these substances. The fact that these other resistances are greater for S12, P12, and P13 than for tertiary amines of similar chain length (T13 and T14) may be due to the higher p*K*_a_ (Table S3) of the former giving a smaller neutral fraction in water.

The experiment at pH 6.2 largely corroborates the dominance of the blood resistance for T10; a 10-fold increase in the overall resistance is matched by a corresponding increase in the blood resistance (Figure 3, right panel). For the other three chemicals tested the overall resistance is also higher at the lower pH, but the water resistance is unaffected. This indicates that the membrane or cytosol resistance is greater at lower pH and now dominates for all of these chemicals. Again, this could be related to the smaller neutral fraction of the chemicals at lower pH. Note that the trend of increasing *k*_U_ with increasing pH observed between pH 6.2 and 7.6 is not expected to continue above pH 7.6 because the water resistance imposes a ceiling on *k*_U_. The experimental results for Q10 and Q14 show that the systemic uptake of the charged form of the amines is strongly inhibited.

### Mechanistic Assessment of Alkylamine Elimination

The rate constant for elimination across the gills, *k*_2_, can be defined as follows:
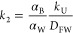
1where α_B_ and α_W_ are the neutral fractions of the chemical in the blood and in the water at the gill surface, respectively, and *D*_FW_ is the fish-water distribution ratio of the chemical at the pH of blood (8.1) (see Text S3). Using the measured *k*_U_ and the values of α_W_, α_B_, and *D*_FW_ estimated for the model, *k*_2_ was calculated (Table 2).

We first return to the pH dependence of *k*_T_ presented above. The modeled pH dependence of *k*_2_ was similar to the measured pH dependence of *k*_T_ for T10 and T13, providing evidence that elimination of these compounds was dominated by gill ventilation. For T10 the pH dependence was weak, with *k*_T_ increasing by a factor of 1.7 between pH 7.6 and pH 6.2. This is consistent with the blood resistance controlling transport across the gills, as in this case *k*_T_ is independent of α_W_ (eq S4.4 and S4.9). For T13 *k*_T_ displayed a stronger pH dependence, increasing by a factor of 2.8 at pH 6.2. However, this is still far less than the change in α_W_ between the pH 7.6 and pH 6.2 experiments (a factor 14). This intermediate pH dependency for T13 is consistent with the conclusion above that the water resistance was dominant at pH 7.6 while the membrane or cytosol resistance dominated at pH 6.2. The water resistance for elimination is proportional to α_W_ (eq S4.7), while the membrane and cytosol resistances for elimination would not be expected to be influenced by gill water pH.

For P12 and P16 there were large discrepancies between the pH dependency of *k*_T_ and *k*_2_. For P12 the pH dependency of *k*_T_ (a factor 6.3) exceeded that of *k*_2_ (factor 2.5). We have no mechanistic explanation for this and note that it may be partly attributable to the high uncertainty in measured *k*_U_ (used to estimate *k*_2_) and *k*_T_ for this substance. In contrast, P16 displayed no pH dependency for *k*_T_ but a strong dependency for *k*_2_ (factor 7). One explanation could be that elimination of P16 was not dominated by gill ventilation, but rather by biotransformation or another elimination process.

Comparing *k*_2_ and *k*_T_ for the same substance, *k*_2_ exceeded *k*_T_ by a factor of ∼5 for T9 and T10, while the values were quite similar for T13, T14, and S12, and *k*_2_ was less than *k*_T_ by a factor of ∼10 for P12 and P13 and ∼100 for S16 and P16. The elimination rate constant for one process cannot be larger than the elimination rate constant for the whole system, so the large exceedance of *k*_T_ by *k*_2_ for T9 and T10 indicates that there are weaknesses in either the observations or the model as represented by eq 1. The data for the uptake and elimination kinetics of T9 and T10 were of good quality (see Figures S6 and S7), and errors in *k*_T_ and *k*_U_ will be highly correlated and thus unlikely to explain a major portion of the exceedance. Another possibility is that α_B_ is overestimated because blood pH was set too high. The pH used in the model, 8.1, was based on measurements conducted in the blood of rainbow trout.^23^ A further possibility is that α_W_ was underestimated because the pH in water was set too low. The pH exiting the gills was estimated to be reduced by 0.5 pH units during the pH 7.6 experiment as a result of CO_2_ exhalation by the fish and the limited buffering capacity of the aquarium water. An overestimation of this effect would result in an overestimation of *k*_2_. However, for the pH 6.2 experiment the modeled pH reduction in the gills was minimal, but *k*_2_ still exceeded *k*_T_ by a factor of 4 for T10 (Table 2). A final possibility is that *D*_FW_ was underestimated. *D*_FW_ was calculated from measured membrane lipid–water partition coefficients assuming a fish membrane lipid content of 1% and a correction factor of 3.5 to account for the additional sorption of cations to anionic phospholipids present in membranes (see Text S3).^35,36^ More research is required to better understand *D*_FW_.

A value of *k*_2_/*k*_T_ ≈ 1 indicates that gill ventilation is a major elimination process. Our observations suggest that this was the case for T13, T14, and S12. However, for P12, P13, P16, and S16 we observed *k*_2_/*k*_T_ ≪1, which suggests that another elimination process dominated. Due to the high uncertainty in the rate constants for P12 and P13, we draw no conclusions for these substances. However, for P16 and S16 the results convincingly show that another process dominates as the data quality is better and *k*_T_ is 30–100 times larger than *k*_2_ (Table 2). One possible conclusion is that P16 and S16 (and possibly P12 and P13) were eliminated primarily via biotransformation. However, in vitro biotransformation tests using S9 assays have shown no transformation of primary alkylamines. Furthermore, shorter chained secondary and tertiary alkylamines for which our results indicate limited biotransformation are readily transformed in the S9 assay.^27,37^ More research is required to resolve this apparent contradiction.

### Mechanistic Assessment of QAC Bioconcentration

Apart from traces in blood, QACs were found only in the gills and skin/mucus of rainbow trout that had been exposed to the test chemicals for 7 days.^32^ This indicates that the accumulation of these chemicals occurred predominantly via sorption to surface tissue, not via transport across the gills followed by systemic distribution and accumulation in internal tissues/organs. The uptake kinetics of Q14 were nearly linear over the 14 d exposure phase (Figure S6). There was little evidence of a two-phase behavior during uptake; initial rapid sorption accounted for <5% of the cumulative uptake over 14 d. This suggests that uptake of Q14 can be conceptualized as transfer of the charged molecule from water to a single storage reservoir. The lack of pH dependence of the elimination rate suggests that the dominant mass transfer resistance is located in the tissue and not in the water. Two consequences of this are that the uptake rate constant would be proportional to the tissue/water partition coefficient of the (charged) chemical because uptake would be driven by the concentration gradient in the tissue, and elimination rate constants would be the same for all chemicals as long as their diffusion coefficients in the sorbed phase were similar. More experiments would be required to determine whether the reservoir is well mixed and separated from the water by a diffusion barrier, or whether the chemical diffuses gradually into an unmixed reservoir.

A significant fraction of the alkylamines was also found in the gills and skin/mucus in the tissue distribution experiment,^31^ which indicates that the processes governing Q14 accumulation also influence the accumulation of the alkylamines. Of the alkylamines, the fraction in gills and skin/mucus after the 7 d exposure was greatest for P16 and S16 (73% and 71%, respectively). Some features of the behavior of P16 and S16 were also consistent with the mechanistic framework for Q14 bioconcentration. For instance, *k*_U_ and *k*_T_ for P16 were both largely independent of pH; *k*_U_ for Q14, P16, and S16 were approximately proportional to *D*_MLW_ (Table S25); *k*_T_ was similar for Q14, P16, and S16 despite their very different BCFs (Figure 1). It is clear that the behavior of P16 and S16 is different from the shorter chained amines, and hence the behavior of the shorter chained amines cannot be readily extrapolated to the longer chained substances. More research is required to explore the influence of pH and surface sorption on the bioconcentration of alkylamines with chain lengths of C_16_ and longer, particularly because the observed BCFs for P16 and S16 are well above the regulatory thresholds and therefore indicate a concern if such chemicals have high emissions into the environment.

## Supporting Information Available

Text S1, quality assurance of the analytical methods; Text S2, model fitting; Text S3, mechanistic model of amine bioconcentration; Text S4, estimation of the sorbed fraction of the surfactants in aquarium water; Text S5, estimation of pH at the gill surface; Table S1, cationic surfactants with tonnage >1000/y in EU; Table S2, chemicals; Table S3, test chemical properties; Table S4, solvents; Table S5, sampling schedule; Table S6, precision of water analysis; Table S7, blanks from water analysis; Table S8, precision of analysis of raw fish extracts; Table S9, precision of analysis of fish homogenate; Table S10, recovery of spiked test chemicals during analysis of fish; Table S11, LOQ for analysis of fish; Table S12, alkalinity and pH in the aquaria during the experiments; Table S13, total organic carbon in the aquaria during the experiments; Tables S14–S22, concentrations of test chemicals in experiments; Table S23, inter-individual variability of test chemical concentrations in fish; Table S24, demethylation products of test chemicals found in 3 fish; Table S25, comparison of *k*_U_ and *k*_U_/DMLW for Q14, S16, and P16; Figure S1, relative quantity extracted from fish homogenate in successive batch extractions at different temperatures; Figure S2, pH during the MIX1pH experiment; Figures S3–S5, semilogarithmic plots of test chemical elimination and experiments; and Figures S6–S8, plots of chemical uptake and elimination in the experiments (PDF)

## References

[ref1] Giolando S. T.; Rapaport R. A.; Larson R. J.; Federle T. W.; Stalmans M.; Masscheleyn P. Environmental fate and effects of DEEDMAC: A new rapidly biodegradable cationic surfactant for use in fabric softeners. Chemosphere 1995, 30, 1067–1083. 10.1016/0045-6535(95)00005-S.

[ref2] Fredell D. L. Biological Properties and Applications of Cationic Surfactants. In Cationic Surfactants: Analytical and Biological Evaluation; Cross J., Singer E. J., Eds.; CRC Press: Boca Raton, 1994; Vol. 53, pp 31–60.

[ref3] Krogh K.; Mogensen B.; Halling-Sörensen B.; Cortés A.; Vejrup K.; Barceló D. Analysis of alcohol ethoxylates and alkylamine ethoxylates in agricultural soils using pressurised liquid extraction and liquid chromatography-mass spectrometry. Anal. Bioanal. Chem. 2003, 376, 1089–1097. 10.1007/s00216-003-2062-3. 12904945

[ref4] Praus P.; Turicová M.; Študentová S.; Ritz M. Study of cetyltrimethylammonium and cetylpyridinium adsorption on montmorillonite. J. Colloid Interface Sci. 2006, 304, 29–36. 10.1016/j.jcis.2006.08.038. 17007866

[ref5] Gromaire M. C.; Van de Voorde A.; Lorgeoux C.; Chebbo G. Benzalkonium runoff from roofs treated with biocide products – *In situ* pilot-scale study. Water Res. 2015, 81, 279–287. 10.1016/j.watres.2015.05.060. 26081434

[ref6] Kahrilas G. A.; Blotevogel J.; Stewart P. S.; Borch T. Biocides in hydraulic fracturing fluids: A critical review of their usage, mobility, degradation, and toxicity. Environ. Sci. Technol. 2015, 49, 16–32. 10.1021/es503724k. 25427278

[ref7] Pati S. G.; Arnold W. A. Comprehensive screening of quaternary ammonium surfactants and ionic liquids in wastewater effluents and lake sediments. Environ. Sci. Process. Impacts 2020, 22, 430–441. 10.1039/C9EM00554D. 32003378

[ref8] Weisbrod A. V.; Woodburn K. B.; Koelmans A. A.; Parkerton T. F.; McElroy A. E.; Borgå K. Evaluation of bioaccumulation using in vivo laboratory and field studies. Integr. Environ. Assess. Manage. 2009, 5, 598–623. 10.1897/IEAM_2009-004.1. 19552500

[ref9] Timmer N.; Droge S. T. J. Sorption of cationic surfactants to artificial cell membranes: comparing phospholipid bilayers with monolayer coatings and molecular simulations. Environ. Sci. Technol. 2017, 51, 2890–2898. 10.1021/acs.est.6b05662. 28187261 PMC5343551

[ref10] OECD., Test No. 305: Bioaccumulation in Fish: Aqueous and Dietary Exposure, OECD Guidelines for the Testing of Chemicals, Section 3; OECD Publishing: Paris, 2012 10.1787/9789264185296-en.

[ref11] Groothuis F. A.; Timmer N.; Opsahl E.; Nicol B.; Droge S. T. J.; Blaauboer B. J.; Kramer N. I. Influence of in vitro assay setup on the apparent cytotoxic potency of benzalkonium chlorides. Chem. Res. Toxicol. 2019, 32, 1103–1114. 10.1021/acs.chemrestox.8b00412. 31012305 PMC6584903

[ref12] Timmer N.; Scherpenisse P.; Hermens J. L. M.; Droge S. T. J. Evaluating solid phase (micro-) extraction tools to analyze freely ionizable and permanently charged cationic surfactants. Anal. Chim. Acta 2018, 1002, 26–38. 10.1016/j.aca.2017.11.051. 29306411

[ref13] Arnot J. A.; Gobas F. A. P. C. A review of bioconcentration factor (BCF) and bioaccumulation factor (BAF) assessments for organic chemicals in aquatic organisms. Environ. Rev. 2006, 14, 257–297. 10.1139/a06-005.

[ref14] Krop H.; de Voogt P. Bioconcentration Factors of Surfactants in Seawater; IBED/IVAM: Amsterdam, 2007 https://hdl.handle.net/11245/1.278502.

[ref15] ECHA. Assessment Report Didecyldimethylammonium chloride Product-type 8 (Wood preservative), June 2015, Italy. p 472 in Data_008.pdf https://echa.europa.eu/documents/10162/fc7cb9c2-924f-e66e-e26b-6cadc4eb22dd.

[ref16] ECHA. Assessment Report Alkyl (C12–16) dimethylbenzyl ammonium chloride Product-type 8 (Wood preservative) June 2015, Italy. p 466 in Data_011.pdf https://echa.europa.eu/documents/10162/6ec12942-2312-d67b-176f-df9d43fa93f3.

[ref17] Valls M.; Fernandez P.; Bayona J. M. M. Fate of cationic surfactants in the marine environment, I. Bioconcentration of long-chain alkylnitriles and trialkylamines. Chemosphere 1989, 19, 1819–1827. 10.1016/0045-6535(89)90005-2.

[ref18] Mackay D. Correlation of bioconcentration factors. Environ. Sci. Technol. 1982, 16, 274–276. 10.1021/es00099a008. 22257252

[ref19] Dassuncao C.; Pickard H.; Pfohl M.; Tokranov A. K.; Li M.; Mikkelsen B.; Slitt A.; Sunderland E. M. Phospholipid levels predict the tissue distribution of poly- and perfluoroalkyl substances in a marine mammal. Environ. Sci. Technol. Lett. 2019, 6, 119–125. 10.1021/acs.estlett.9b00031. 33283018 PMC7713714

[ref20] Clark K. E.; Gobas F. A. P. C.; Mackay D. Model of organic chemical uptake and clearance by fish from food and water. Environ. Sci. Technol. 1990, 24, 1203–1213. 10.1021/es00078a008.

[ref21] Arnot J. A.; Mackay D.; Bonnell M. Estimating metabolic biotransformation rates in fish from laboratory data. Environ. Toxicol. Chem. 2008, 27, 341–351. 10.1897/07-310R.1. 18348640

[ref22] Erickson R. J.; McKim J. M.; Lien G. J.; Hoffman A. D.; Batterman S. L. Uptake and elimination of ionizable organic chemicals at fish gills: I. Model formulation, parameterization, and behavior. Environ. Toxicol. Chem. 2006, 25 (6), 1512–1521. 10.1897/05-358R.1. 16764469

[ref23] Erickson R. J.; McKim J. M.; Lien G. J.; Hoffman A. D.; Batterman S. L. Uptake and elimination of ionizable organic chemicals at fish gills: II. Observed and predicted effects of ph, alkalinity, and chemical properties. Environ. Toxicol. Chem. 2006, 25 (6), 1522–1532. 10.1897/05-359R.1. 16764470

[ref24] Mansy S. S. Membrane transport in primitive cells. Cold Spring Harbor Perspect. Biol. 2010, 2, a002188 10.1101/cshperspect.a002188. PMC2908771 20679338

[ref25] Bittner L.; Klüver N.; Henneberger L.; Mühlenbrink M.; Zarfl C.; Escher B. Combined ion-trapping and mass balance models to describe the pH-dependent uptake and toxicity of acidic and basic pharmaceuticals in zebrafish embryos (*Danio rerio*). Environ. Sci. Technol. 2019, 53, 7877–7886. 10.1021/acs.est.9b02563. 31177773

[ref26] Pritchard J. B.; Miller D. S. Renal secretion of organic anions and cations. Kidney Int. 1996, 49, 1649–1654. 10.1038/ki.1996.240. 8743470

[ref27] Chen Y.; Hermens J. L. M.; Jonker M. T. O.; Arnot J. A.; Armitage J. M.; Brown T.; Nichols J. W.; Fay K. A.; Droge S. T. J. Which molecular features affect the intrinsic hepatic clearance rate of ionizable organic chemicals in fish?. Environ. Sci. Technol. 2016, 50, 12722–12731. 10.1021/acs.est.6b03504. 27934284

[ref28] Krause S.; Goss K.-U. In vitro- in vivo extrapolation of hepatic metabolism for different scenarios - a toolbox. Chem. Res. Toxicol. 2018, 31, 1195–1202. 10.1021/acs.chemrestox.8b00187. 30259739

[ref29] Nichols J. W.; Huggett D. B.; Arnot J. A.; Fitzsimmons P. N.; Cowan-Ellsberry C. E. Toward improved models for predicting bioconcentration of well-metabolized compounds by rainbow trout using measured rates of in vitro intrinsic clearance. Environ. Toxicol. Chem. 2013, 32 (7), 1611–1622. 10.1002/etc.2219. 23504707

[ref30] Armitage J. M.; Arnot J. A.; Wania F.; Mackay D. Development and evaluation of a mechanistic bioconcentration model for ionogenic organic chemicals in fish. Environ. Toxicol. Chem. 2013, 32, 115–128. 10.1002/etc.2020. 23023933

[ref31] Kierkegaard A.; Chen C.; Armitage J. M.; Arnot J. A.; Droge S.; McLachlan M. S. Tissue distribution of several series of cationic surfactants in rainbow trout (*Oncorhynchus mykiss*) following exposure via water. Environ. Sci. Technol. 2020, 54, 4190–4199. 10.1021/acs.est.9b07600. 32062967 PMC7343282

[ref32] Barber M. C. A review and comparison of models for predicting dynamic chemical bioconcentration in fish. Environ. Toxicol. Chem. 2003, 22, 1963–1992. 10.1897/02-468. 12959521

[ref33] Adolfsson-Erici M.; Åkerman G.; McLachlan M. S. Internal benchmarking improves precision and reduces animal requirements for determination of fish bioconcentration factors. Environ. Sci. Technol. 2012, 46, 8205–8211. 10.1021/es301700e. 22809237

[ref37] Droge S. T. J.; Armitage J. M.; Arnot J. A.; Fitzsimmons P. N.; Nichols J. W. Biotransformation potential of cationic surfactants in fish assessed with rainbow trout liver S9 fractions. *Environ. Toxicol. Chem.* (submitted). 10.1002/etc.5189 PMC9187044 34379820

[ref34] Ebert A.; Hannesschlaeger C.; Goss K.-U.; Pohl P. Passive permeability of planar lipid bilayers to organic anions. Biophys. J. 2018, 115, 1931–1941. 10.1016/j.bpj.2018.09.025. 30360927 PMC6303230

[ref35] Schmitt W. General approach for the calculation of tissue to plasma partition coefficients. Toxicol. In Vitro 2008, 22, 457–467. 10.1016/j.tiv.2007.09.010. 17981004

[ref36] Elsayed M. M. A.; Vierl U.; Cevc G. Accurate potentiometric determination of lipid membrane–water partition coefficients and apparent dissociation constants of ionizable drugs: Electrostatic corrections. Pharm. Res. 2009, 26, 1332–1343. 10.1007/s11095-009-9842-1. 19283456

