# Supplementary material for: Bioconcentration of Several Series of Cationic Surfactants in Rainbow Trout
Source: Environ Sci Technol. 2021 Jun 16;55(13):8888–97. doi: 10.1021/acs.est.1c02063 (PMC8277129; doi:10.1021/acs.est.1c02063)
Supplement: Supplementary file 1 — es1c02063_si_001.pdf [file es1c02063_si_001.pdf]

SUPPORTING INFORMATION
for the paper

Bioconcentration of several series of cationic surfactants in rainbow trout

Amelie Kierkegaard[1], Marcus Sundbom[1], Bo Yuan[1], James M. Armitage[2], Jon A. Arnot[3,4], Steven T.J. Droge[5,6], Michael S. McLachlan[1*]

[1]Department of Environmental Science, Stockholm University

[2]AES Armitage Environmental Sciences, Inc.

[3]ARC Arnot Research and Consulting Inc.

[4]Department of Physical and Environmental Sciences, University of Toronto Scarborough

[5]Institute for Biodiversity and Ecosystem Dynamics (IBED), University of Amsterdam

[6]Dutch Board for the Authorisation of Plant Protection Products and Biocides (Ctgb)

E-mail contact: michael.mclachlan@aces.su.se

Table of Contents

**Text**		
S1	Quality assurance of the analytical methods	S3
S2	Model fitting	S4
S3	A mechanistic model of amine bioconcentration	S5
S4	Estimation of the sorbed fraction of the surfactants in aquarium water	S7
S5	Estimation of pH at the gill surface	S8
**Tables**		
S1	Cationic surfactants with tonnage >1000/y in EU	S10
S2	Chemicals	S12
S3	Test chemical properties	S13
S4	Solvents	S14
S5	Sampling schedule	S15
S6	Precision of water analysis	S16
S7	Blanks from water analysis	S16
S8	Precision of analysis of raw fish extracts	S16
S9	Precision of analysis of fish homogenate	S17
S10	Recovery of spiked test chemicals during analysis of fish	S17
S11	LOQ for analysis of fish	S17
S12	Alkalinity and pH in the aquaria during the experiments	S18
S13	Total organic carbon (TOC) in the aquaria during the experiments	S19
S14	Concentrations of test chemicals in water / MIX1 experiment	S20
S15	Concentrations of test chemicals in water / MIX2 experiment	S21
S16	Concentrations of test chemicals in water / MIX1pH experiment	S22
S17	Concentrations of test chemicals in fish / exposure phase of the MIX1 exp't	S24
S18	Concentrations of test chemicals in fish / elimination phase of the MIX1 exp't	S25
S19	Concentrations of test chemicals in fish / exposure phase of the MIX2 exp't	S26
S20	Concentrations of test chemicals in fish / elimination phase of the MIX1 exp't	S27
S21	Concentrations of test chemicals in fish / exposure phase of the MIX2 exp't	S28
S22	Concentrations of test chemicals in fish / elimination phase of the MIX1pH exp't	S29
S23	Inter-individual variability of test chemical concentrations in fish	S30
S24	Demethylation products of test chemicals found in 3 fish	S30
S25	Comparison of k_U and k_U/D_{MLW} for Q14, S16 and P16	S31
**Figures**		
S1	Relative quantity extracted from fish homogenate in successive batch extractions at different temperatures	S32
S2	pH during the MIX1pH experiment	S32
S3	Semilogarithmic plot of test chemical elimination / MIX1 experiment	S33
S4	Semilogarithmic plot of test chemical elimination / MIX2 experiment	S34
S5	Semilogarithmic plot of test chemical elimination / MIX1pH experiment	S35
S6	Plot of chemical uptake and elimination / MIX1 experiment with model fit	S36
S7	Plot of chemical uptake and elimination / MIX2 experiment with model fit	S37
S8	Plot of chemical uptake and elimination / MIX1pH experiment with model fit	S38

<u>Text S1</u>: <u>Quality assurance of the analytical methods</u>

For the water method, the repeatability, quantified as the average relative standard deviation of the 21 sets of triplicate samples collected during the exposure phase, ranged between 1-6% (Table S6). Outliers were rare, accounting for at most 6% of the measurements, and were with one exception positive outliers that impacted several or all of the more hydrophobic analytes in a given sample. We believe that the outliers were caused by the presence of a large particle (e.g., a feces fragment) in the sampled water, and these data were therefore discarded. The concentrations in the method blanks for the water samples were low (Table S7), with the maximum blank seldom exceeding 1% of the target concentration in water.

For fish analysis, method precision was assessed by analyzing inter-batch duplicates for a selection of the samples. When the replication only included sample clean-up and instrumental analysis (i.e., a common extract was used), the mean relative difference between the duplicates ranged from 2% to 7% when the concentrations were above the LOQ (Table S8). When the replication also included the extraction step, the mean relative difference was similar (Table S9). The mean recovery of standards spiked to fish homogenate or raw extract ranged from 92-116% (see Table S10), which provided confidence in the accuracy of the method. Experiments were conducted to test extraction efficiency, and the extracted quantity of test chemical decreased by a factor of 10 between the first and second, and again between the second and third 1 h batch sonication steps (see Figure S1). This indicated that 2 batch extractions were sufficiently exhaustive, and this procedure was chosen.

The test chemical concentrations in the control fish and the fish method blanks were <5 ng g^{-1} in most cases. The LOQ of the method (calculated as 10 × standard deviation of the blank/control fish) ranged from 3-9 ng g^{-1} (Table S11). However, two batches for fish from the exposure phase of the MIX1pH experiment had elevated blank levels of P9, T10 and T13 (39±5, 202±21 and 59±2 ng g^{-1}, respectively). The resulting blank correction resulted in P9 being unquantifiable for this experiment and T10 lying between the LOD and LOQ for 16 of 24 data points during the exposure phase. The data for T10 were used for model fitting despite their higher uncertainty.

Text S2: Model fitting

The data were fit by integrating the equation

$$\frac{dC_F}{dt} = k_U C_W - k_T C_F$$

stepwise from one sampling time point to the next. A function of concentration in water versus time was created from the measurements for both the exposure phase and elimination phase, so no information was lost by averaging the concentrations over the exposure phase or assuming a concentration of zero during elimination. The concentration in water C_W was assumed to be constant between sampling time points and equal to the average of the concentrations measured at the start and the end of the interval (see Tables S14-S16 for the measured data). The concentration in fish at the end of the interval $C_{F(t+1)}$ could be then calculated from the concentration at the beginning of the interval $C_{F(t+1)}$ using

$$C_{F(t+1)} = \left(C_{F(t)} - \frac{k_U}{k_T} C_W\right) e^{-k_T \Delta t} + \frac{k_U}{k_T} C_W$$

The model was fitted simultaneously for k_U and k_T using the solver function in Microsoft Excel. The fitting criterion was to minimize the sum of the normalized residuals ($C_{F(measured)}$ – $C_{F(modeled)}$), whereby the normalization was done to the smaller of the measured and modeled concentrations:

$$fitting\ criterion = minimize \sum \frac{|(C_{F(measured)} - C_{F(modeled)})|}{\text{MIN}\ (C_{F(measured)}, C_{F(modeled)})}$$

By normalizing the residuals, the bias associated with the magnitude of the concentration is eliminated. The fitting was done using $C_{F(measured)}$ for individual fish. All time points were included for which all three fish were above the LOQ, with the exception of the first 3 time points during the exposure phase. They were not included in the fitting procedure as there was evidence of a small, rapidly responding compartment that led to large relative errors at the beginning of the model simulation, errors which then dominated the fitting criterion. This small, rapidly responding compartment may have been skin mucus (Kierkegaard et al., 2020). For P12 in the MIX1pH experiment, three fish were removed as outliers; these fish had concentrations that were an order of magnitude higher than the others.

Text S3: A mechanistic model of amine bioconcentration

The one-box mass balance model of chemical bioconcentration in fish considers three mass transfer processes: diffusive uptake across the gills, diffusive elimination across the gills, and biotransformation (Barber, 2003). The governing mass balance equation is:

$$M\frac{dC_F}{dt} = \frac{1}{R_{W\rightarrow B}}A_G C_W - \frac{1}{R_{B\rightarrow W}}\frac{A_G}{D_{FW}}C_F - Mk_B C_F \quad \text{(S4.1)}$$

where M is fish mass (kg), A_G is the surface area of the gills (m^2), C_F is concentration of the chemical in the fish (mol kg^{-1}), C_W is the concentration of freely dissolved chemical in the water (mol m^{-3}), D_{FW} is the distribution ratio of the chemical (both charged and neutral forms) between the fish and water (m^3 kg^{-1}), $R_{W\rightarrow B}$ and $R_{B\rightarrow W}$ are the overall resistances for transport across the gills from water to blood and blood to water, respectively (h m^{-1}), and k_B is the first order rate constant for transformation of the chemical in the fish (h^{-1}) (Erickson et al., 2006). Comparing with the rate constant model used for bioconcentration assessment (OECD, 2012)

$$\frac{dC_F}{dt} = k_U C_W - k_T C_F \quad \text{(S4.2)}$$

the uptake rate constant k_U is defined as:

$$k_U = \frac{1000 A_G}{R_{W\rightarrow B} M} \quad \text{(S4.3)}$$

where 1000 is a conversion factor from m^3 to L, and the elimination rate constant k_T is defined as:

$$k_T = \frac{1}{R_{B\rightarrow W}}\frac{A_G}{M D_{FW}} + k_B = k_2 + k_B \quad \text{(S4.4)}$$

where k_2 is the rate constant for gill elimination. M was set to the average mass of the fish in the experiment (0.01094 kg). A_G was calculated for a fish of this mass using the correlation for rainbow trout in Hughes (1984). D_{FW} was calculated as 1% of the membrane-water distribution coefficient ($D_{MLW,T}$, m^2 kg^{-1}), since fish contain approximately 1% membrane lipids by mass (Hendriks et al., 2005). $D_{MLW,T}$ was based on measurements of the distribution ratio between water and a neutral phospholipid experimental model (D_{MLW}, m^2 kg^{-1}, see Table S3). The selection of $D_{MLW,T}$ as the basis for D_{FW} was based on:

(i) the availability of sufficient D_{MLW} values to allow for extrapolations to all test compounds (Timmer and Droge, 2017) and insufficient data on other tissue components;
(ii) measurements showing for one cationic surfactant that partitioning to membrane lipids is greater than to structural muscle protein (D_{SPW}) (C_8-benzalkonium log D_{MLW} is 3.11-3.63; (Timmer and Droge, 2017, Bittermann et al., 2014), log D_{SPW} is 1.4 (Henneberger et al., 2016));
(iii) D_{SPW} for several cations being comparable to albumin blood protein (D_{BPW}) (Henneberger et al., 2016).

Membranes also contain anionic phospholipids which enhance the sorption of cations (Elsayed et al., 2009). This effect was accounted for using a regression developed by Schmitt:

$$D_{MLW,T} = D_{MLW}(1 + 20 f_{PL-}) \quad \text{(S4.5)}$$

where f_{PL-} is the fraction of anionic phospholipids in the membrane. f_{PL-} was set to 0.175 based on data reported in Schmitt (2008).

$R_{W\to B}$ and $R_{B\to W}$ can be treated as the sum of 4 resistances acting in series: water (R_W), membrane (R_M), cytosol (R_C) and blood (R_B). The water and blood flow resistances were assumed to be flow governed and estimated as

$$R_{W,W\to B} = \frac{A_G}{Q_W} \quad \text{(S4.6)}$$

$$R_{W,B\to W} = \frac{A_G}{Q_W}\left[\frac{\alpha_W}{\alpha_B}\right] \quad \text{(S4.7)}$$

$$R_{B,W\to B} = \frac{A_G}{Q_B(1 + f_{PL-B}D_{MLW})}\left[\frac{\alpha_B}{\alpha_W}\right] \quad \text{(S4.8)}$$

$$R_{B,B\to W} = \frac{A_G}{Q_B(1 + f_{PL-B}D_{MLW})} \quad \text{(S4.9)}$$

where Q_W and Q_B are the flow rates through the gills of water and blood, respectively ($m^3\ h^{-1}$), f_{PL-B} is the fraction of polar lipids in the blood, and α_W and α_B are the neutral fraction of the chemical in the water and blood, respectively (Erickson et al., 2006). The extra term in the denominator of the blood resistance equations accounts for the increased transport capacity due to sorption of the chemicals to membrane lipids present in the blood. No influence of sorption on transport capacity was included for water. The quotient of the neutral fractions is a consequence of only the neutral fraction of the chemical passing through the membrane. It accounts for the change in the transport capacity of the distant medium with respect to the reference medium (water in the case of $R_{W\to B}$ and blood in the case of $R_{B\to W}$) as a result of the change in pH.

We did not find sufficient information to construct a well-constrained model of the resistances for membrane (R_M), and cytosol (R_C) for cationic surfactants. They are unknown in the model.

The model was parameterized for the conditions used in the experiment. Q_W was estimated to be $2.80\times10^{-4}\ m^3\ h^{-1}$ based on an average fish mass of 10.9 g, water temperature of 10 °C and an O_2 saturation of 95% using the method of Arnot and Gobas (2004). Q_B was $2.45\times10^{-5}\ m^3\ h^{-1}$ using the method of Erickson et al. (2006). f_{PL-B} was set equal to 0.007, 50% of the lipid of rainbow trout blood reported by Bertelsen et al. (1998). The pH of water at the gill surface, and hence α_W, varied along the gill lamella (see Text S5). Therefore, the gill was treated as 10 sequential segments. The resistances were determined for each segment using the water pH in that segment (see Text S5). These 10 parallel resistances were then combined to give an overall resistance.

The water flow and blood flow resistances for elimination each differ from the corresponding resistance for uptake by a constant, the ratio of the neutral fraction of the chemical in water and blood α_W/α_B (Eq. S4.7 and S4.8). This is also true for membrane and cytosol resistances when the uptake of the charged form is negligible (Erickson et al., 2006). Consequently, the relative contributions of the different resistances are the same during uptake and elimination, and the overall resistance for gill elimination $R_{B\to W}$ can be calculated from the overall resistance for gill uptake $R_{W\to B}$:

$$R_{B\to W} = \frac{\alpha_W}{\alpha_B} R_{W\to B} \quad \text{(S4.10)}$$

where α_W/α_B describes the effect of ion trapping on the resistance. Substituting into Eq. 4.4, it follows that the elimination rate constant can be defined as follows:

$$k_2 = \frac{\alpha_B\ k_U}{\alpha_W D_{FW}} \quad \text{(S4.11)}$$

Text S4: Estimation of the fraction of the surfactants in aquarium water during the exposure experiment that was sorbed to organic material

The organic carbon-water distribution coefficient D_{OC} [L kg^{-1}] was estimated with a model derived from a large set of measurements of distribution of cationic surfactants between water and standard soil organic matter in medium with a divalent cation content of 5 mM Ca^{2+} (Droge and Goss, 2013):

$$\log D_{OC} = 1.70\, V_x + 0.397\, NA_i - 0.725$$

where V_x is McGowan's molecular volume and NA_i is the number of H-atoms attached to the charged N moiety.

The D_{OC} value for protonated amines and QAC was used together with the average TOC concentration in the aquaria (5.5 mg L^{-1}) to estimate the freely dissolved fraction. The results are summarized in this table, with freely dissolved fractions calculated (with TOC in units of kg L^{-1}):

$$\text{Freely dissolved fraction} = \frac{1}{1 + (D_{OC} \cdot TOC)}$$

Chemical	V_x	NA_i	log D_{OC}	Freely dissolved fractions
P9	1.4765	3	2.98	0.99
P12	1.9207	3	3.73	0.97
P13	2.0401	3	3.93	0.96
P16	2.4628	3	4.65	0.80
S12	2.0401	2	3.54	0.98
S16	2.6037	2	4.50	0.85
T9	1.7583	2	3.06	0.99
T10	1.8992	1	2.90	1.00
T13	2.3219	1	3.62	0.98
T14	2.4628	1	3.86	0.96
Q10	2.0831	0	2.82	1.00
Q14	2.6467	0	3.77	0.97

The freely dissolved fraction was smallest for P16, for which 80% of the amount in water was estimated to be freely dissolved.

At a 10 times lower water hardness than anticipated by 5 mM Ca^{2+}, which may be more reflective of the aquarium water, the logD_{OC} is expected to increase by 0.3 log units. For the strongest sorbing amine P16 an estimated logD_{OC} (at 0.5 mM Ca^{2+}) of 4.95, this could result in a further reduction of the freely dissolved fraction to 0.67. The apparent aqueous concentration of P16 to which the fish were exposed may thus be overestimated by 33%, which would increase the fitted BCF proportionally.

Text S5: Estimation of pH at the gill surface

The pH at the surface of the gills was calculated using the following system of 5 equations:

$$\frac{[H^+][HCO_3^-]}{[H_2CO_3]} = K_1 \tag{1}$$

$$\frac{[H^+][CO_3^{2-}]}{[HCO_3^-]} = K_2 \tag{2}$$

$$alk = 2 \times [CO_3^{2-}] + [HCO_3^-] \tag{3}$$

$$DIC = [H_2CO_3] + [HCO_3^-] + [CO_3^{2-}] \tag{4}$$

$$pH = -log([H^+]) \tag{5}$$

where alk is alkalinity and DIC is total dissolved inorganic carbon. The dissociation constants K_1 and K_2 were estimated from water temperature according to equations 9.23 and 9.24 in

http://www-naweb.iaea.org/napc/ih/documents/global_cycle/vol%20I/cht_i_09.pdf

The measured pH and alkalinity in bulk water were used to solve for $[H^+]$, $[HCO_3^-]$, $[CO_3^{2-}]$, $[H_2CO_3]$ and DIC. For the water in the gill lamella, the carbon added to the water via respiration (0.0002 mol L^{-1} based on estimated oxygen demand and gill ventilation rate for a 10 g fish at 10 °C (Arnot et al. 2008, Armitage et al. 2013)) was added to DIC, and using this new DIC and the alkalinity the 5 equations were resolved for $[H^+]$, $[HCO_3^-]$, $[CO_3^{2-}]$, $[H_2CO_3]$ and pH. The pH gradient along the lamella was approximated by dividing the lamella into 10 segments and assuming that the CO_2 excretion rate was uniform along the length of the lamella. The resulting gradient of pH along the lamella is shown below.

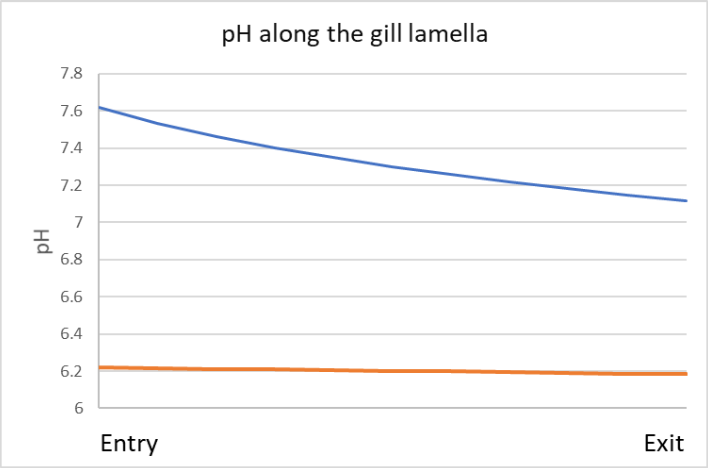

References for Texts S2- S5

Armitage J. M.; Arnot, J. A.; Wania, F.; Mackay, D. Development and evaluation of a mechanistic bioconcentration model for ionogenic organic chemicals in fish. *Environ. Toxicol. Chem.* **2013**, *32*, 115-128.

Arnot, J. A.; Gobas, F. A. P. C. A food web bioaccumulation model for organic chemicals in aquatic ecosystems. *Environ. Toxicol. Chem.* **2004**, *23*, 2343-2355.

Arnot, J. A.; Mackay, D.; Bonnell, M. Estimating metabolic biotransformation rates in fish from laboratory data. *Environ. Toxicol. Chem.* **2008**, *27*, 341-351.

Barber, M. C. A review and comparison of models for predicting dynamic chemical bioconcentration in fish. *Environ. Toxicol. Chem.* **2003**, *22*, 1963−1992.

Bertelsen, S. L.; Hoffman, A. D.; Gallinat, C. A.; Elonen, C. M.; Nichols, J. W. Evaluation of log K_{OW} and tissue lipid content as predictors of chemical partitioning to fish tissues. *Environ. Toxicol. Chem.* **1998**, *17*, 1447−1455.

Bittermann, K.; Spycher, S.; Endo, S.; Pohler, L.; Huniar, U.; Goss, K.-U.; Klamt, A. Prediction of phospholipid-water partition coefficients of ionic organic chemicals using the mechanistic model COSMOmic. *J. Phys. Chem. B* **2014**, *118*, 14833-14842.

Droge, S. T. J.; Goss, K.-U. Ion-exchange affinity of organic cations to natural organic matter: Influence of amine type and nonionic interactions at two different pHs. *Environ. Sci. Technol.* **2013**, *47*, 798-806.

Elsayed, M. M. A.; Vierl, U.; Cevc, G. Accurate potentiometric determination of lipid membrane–water partition coefficients and apparent dissociation constants of ionizable drugs: Electrostatic corrections. *Pharm. Res.* **2009**, *26*, 1332-1343.

Erickson, R. J.; McKim, J. M.; Lien, G. J.; Hoffman, A. D.; Batterman, S. L. Uptake and elimination of ionizable organic chemicals at fish gills: I. Model formulation, parameterization, and behavior. *Environ. Toxicol. Chem.* **2006,** *25*, 1512-1521.

Hendriks, A. J.; Traas, T.P.; Huijbregts, M. A. J. Critical body residues linked to octanol-water partitioning, organism composition, and LC50 QSARs: Meta-analysis and model. *Environ. Sci. Technol.* **2005**, *39*, 3226–3236.

Henneberger, L.; Goss, K.-U.; Endo, S. Partitioning of organic ions to muscle protein: Experimental data, modeling, and implications for in vivo distribution of organic ions. *Environ. Sci. Technol.* **2016**, *50*, 7029-7036.

Hughes, G. M. General anatomy of the gills. In Fish Physiology; Hoar, W. S., Randall, D. J., Eds.; Academic: New York, 1984; Vol XA, pp 1−72.

Kierkegaard, A.; Chen, C.; Armitage, J. M.; Arnot, J. A.; Droge, S.; McLachlan, M. S. Tissue distribution of several series of cationic surfactants in rainbow trout (Oncorhynchus mykiss) following exposure via water. *Environ. Sci. Technol.* **2020**, 54, 4190-4199.

OECD. Test No. 305: Bioaccumulation in Fish: Aqueous and Dietary Exposure, OECD Guidelines for the Testing of Chemicals, Section 3; OECD Publishing: Paris, 2012.

Schmitt, W. General approach for the calculation of tissue to plasma partition coefficients. *Toxicol. in Vitro* **2008**, *22*, 457-467.

Table S1. Cationic surfactants with tonnage >1000/y in EU (REACH data aq. surface tension <65 mN/m). Red: BCF likely >2000

**TONNAGE (y^{-1})**	**CAS**	**Amine Type**	**Chain length range ("average chain length")**	**SUBSTANCE NAME**	**SURFACE TENSION**	**REACH Dossier #**	**Examples on BCF info in REACH-dossier** [a]
100000-1000000	1335202-88-4	Q	C16-18 (17)	Esterification products of fatty acids, C16-18 (even numbered) and C18 (unsaturated) with triethanolamine, dimethyl sulphate-quaternized	39.4	15243	Reference to in vivo BCF: DODMAC
10000-100000	68037-95-6	P	C16-18 (17)	Amines, C16-18 and C18-unsatd. Alkyl	23.1	14682	
10000-100000	90640-32-7	P	C16-18 (17)	Amines, C16-18-alkyl	23	14440	
10000-100000	68037-95-6	P	C16-18 (17)	Amines, C16-18 and C18-unsatd. Alkyl	23.9	14682	
10000-100000	90640-32-7	P	C16-18 (17)	Amines, C16-18-alkyl	51.3	14440	
10000-100000	1079184-43-2	Q	C16-18 (17)	Ethanaminium, 2-hydroxy-N-(2-hydroxyethyl)-N,N-dimethyl-, esters (C16-18/C18-unsatd. fatty ac) Cl	37.5	5845	Reference to in vivo BCF: DODMAC
10000-100000	1079184-43-2	Q	C16-18 (17)	Ethanaminium, 2-hydroxy-N-(2-hydroxyethyl)-N,N-dimethyl-, esters (C16-18/C18-unsatd. fatty ac)	<40	5845	
1000-10000	No CAS	P	C9-11 iso (10)	3-((C9-11-iso,C10-rich)alkyloxy)propan-1-amine	30	12430	
1000-10000	124-22-1	P	C12	dodecan-1-amine	23.1	15915	
1000-10000	111-86-4	P	C8	octan-1-amine	44.2	1996	
1000-10000	124-30-1	P	C18	octadecan-1-amine	23.9	14418	
1000-10000	2156592-58-2	P	C12-18 (15)	Amines, C12-18-(even numbered) and C18-(unsaturated) alkyl	24.3	19387	
1000-10000	1213789-63-9	P	C16-18 (17)	(Z)-octadec-9-enylamine, C16-18-(even numbered, saturated and unsaturated)-alkylamines	24.3	16132	
1000-10000	1226892-43-8	P	C18	Fatty acids, C18 (unsaturated), reaction products with diethylenetriamine	34	15906	
1000-10000	1213789-63-9	P	C18	(Z)-octadec-9-enylamine, C16-18-(even numbered, saturated and unsaturated)-alkylamines	51.3	16132	
1000-10000	111-92-2	S	C4x2 (8)	N-butylbutan-1-amine	50.6	13527	
1000-10000	84649-84-3	T	C12-14 (13)	Amines, C12-14-alkyldimethyl	25.2	14216	BCF data waiving: study scientifically not necessary / other information available
1000-10000	68391-04-8	T	C12-18 (15)	Amines, C12-18-alkyldimethyl	30.5	13558	
1000-10000	112-69-6	T	C16	N,N-dimethylhexadecan-1-amine	30.5	14932	
1000-10000	124-28-7	T	C18	dimethyl(octadecyl)amine	32.2	14746	
1000+	71786-60-2	T	C12-18 DEA (15)	Ethanol, 2,2'-iminobis-, N-C12-18-alkyl derivs.	29	14237	BCF estimated by Kow + in vitro S9, with measured log Kow of 0.7
1000+	25307-17-9	T	C18 DEA	2-[(2-hydroxyethyl)[(9E)-octadec-9-en-1-yl]amino]ethan-1-ol	27	14853	
1000+	1218787-30-4	T	C16-18 DEA (17)	unnamed 2,2'-(C16-18 (even numbered) alkyl imino) diethanol	30	14327	BCF estimated by BCFBAF-model, with measured log Kow of 3.6

1000+	1218787-32-6	T	C18 DEA	2,2'-(C16-18 (even numbered, C18 unsaturated) alkyl imino) diethanol	30	14180	
1000-10000	68424-85-1	Q	C12-16 BAC (14)	Quaternary ammonium compounds, benzyl C12-16 (even numbered)-alkyldimethyl chlorides	28.3	13152	BCF estimated by BCFBAF-model, test substance was found to fall in the applicability domain
1000-10000	68391-05-9	Q	C14x2 (28)	N,N-dimethyl-N-tetradecyltetradecan-1-aminium chloride	28	11882	
1000+	No CAS	Q	C16-18 PO (17)	1-Propanaminium, 2-hydroxy-N-(2-hydroxypropyl)-N,N-dimethyl-, esters with fatty acids, C16-18 (even numbered) and C18 unsaturated., Me sulfates (salts)	37.5	13475	Reference to in vivo BCF: DODMAC
1000-10000	1125503-33-4	Q	C12-14 EO (13)	N-(2-hydroxyethyl)-N,N-dimethyl alkyl-C12-14-(even numbered)-1-aminium chloride	24.9	2006	
1000-10000	68909-18-2	Q	C7	Pyridinium, 1-(phenylmethyl)-, ethyl methyl derivs., chlorides	47.9	21246	
**Lower tonnage cations of interest**							
100-1000	139-08-2	Q	C14 BAC	benzyldimethyltetradecylazanium chloride	34.5	22044	Reference to in vivo BCF: C12-16-ADBAC
100-1000	68391-03-7	Q	C12-18 (15)	Quaternary ammonium compounds, C12-C18 (even numbered) alkyltrimethyl chloride	24.04	12150	Reference to in vivo BCF: C12-16-ADBAC
100-1000	1273322-47-6	P	C16-18 (17)	C16-18-(even numbered, C18-unsaturated)-alkylamines acetates	27.8	20913	Reference to in vivo BCF: 1-hexadecanamine
100-1000	94667-33-1	Q	C14x2 EO (28)	(Bardap 26) Poly(oxy-1,2-ethanediyl), .alpha.-[2-(didecylmethylammonio)ethyl]- .omega.- hydroxy-, propanoate (salt)	30.5	15837	Reference to in vivo BCF: DDAC/Bardac22
0-10	210420-85-2	Q	C10x2 (20)	1-Decanaminium,N-decyl-N,N-dimethyl-, hexanedioate(2:1)	32.3		(=DDAC) but no BCF reported

[a] add the dossier-number in this link at X [https://echa.europa.eu/nl/registration-dossier/-/registered-dossier/X] to retrieve the information submitted on Bioaccumulation in the section on Environmental fate & pathways. Text in red indicates that the dossier refers to an in vivo BCF study.

Table S2: Chemicals

Abbr.	Name	CAS #	Supplier	Purity (%)	Internal std. for quantifying
P9	Nonylamine	112-20-9	TCI	>99.5	Q10IS
P12	Dodecylamine	929-73-7	Acros	>98.5	Q10IS
P13	Tridecylamine	2869-34-3	TCI	>98	Q14IS
P16	Hexadecylamine	143-27-1	Sigma-Aldrich	>98.5	P16IS
S12	N-methyldodecylamine	7311-30-0	Sigma-Aldrich	97	Q10IS
S16	N-methylhexadecylamine	13417-08-8	Sigma-Aldrich	95	P16IS
T9	N,N-dimethylnonylamine	17373-27-2	Sigma-Aldrich	97	Q10IS
T10	N,N-dimethyldecylamine	1120-24-7	TCI	>93	Q10IS
T13	N,N-dimethyltridecylamine	17373-29-4	Sigma-Aldrich	>97	Q14IS
T14	N,N-dimethyltetradecylamine	112-75-4	Sigma-Aldrich	>95	Q14IS
Q10	N,N,N-trimethyl-1-decylammonium	2082-84-0	Sigma-Aldrich	>98	Q10IS
Q14	N,N,N-trimethyl-1-tetradecylammonium	4574-04-3	Sigma-Aldrich	>98	Q14IS
P16IS	D_{33}-Hexadecylamine	1191245-82-5	QMX-Laboratories	98	
Q10IS	D_{21}-N,N,N-trimethyl-1-decylammonium	2082-84-0	QMX-Laboratories	98	
Q14IS	D_{29}-N,N,N-trimethyl-1-tetradecylammonium	95523-73-2	QMX-Laboratories	98	

Table S3: Test chemical properties

Abbr.	pKa[$]	Log D_{OC}[§]	Log $D_{MLW,C}$[#]	SMILES
P9	10.6	2.95	3.72	CCCCCCCCCN
P12	10.6	3.63	5.58	CCCCCCCCCCCCN
P13	10.6	3.81	6.16	CCCCCCCCCCCCCN
P16	10.6	4.46	7.90	CCCCCCCCCCCCCCCCN
S12	10.8	3.49	5.39	CCCCCCCCCCCCNC
S16	10.8	4.35	7.71	CCCCCCCCCCCCCCCCNC
T9	10	3.06	3.07	CCCCCCCCCN(C)C
T10	10	2.96	3.65	CCCCCCCCCCN(C)C
T13	10	3.60	5.88	CCCCCCCCCCCCCN(C)C
T14	10	3.82	6.46	CCCCCCCCCCCCCCN(C)C
Q10		2.92	3.34	CCCCCCCCCC[N+](C)(C)C
Q14		3.78	5.46	CCCCCCCCCCCCCC[N+](C)(C)C

[§] organic carbon-water distribution ratio, calculated according to Droge and Goss (2013) *Environ. Sci. Technol.* 47, 798-806.
[#] membrane-water partition coefficient of the charged form, from Timmer and Droge (2017) *Environ. Sci. Technol.* 51, 2890–2898, extrapolated for P13, P16, S16, T13, T14.
[$] pKa values derived/reported for different alkylamine type analogues. The selected value is expected to be independent of chain length for all longer chain homologues, using a weighted value obtained from the different sources with more focus on experimental data:

	**Algorithm / simulated data**			**Experimental reference data (25°C)**	
	Chem Axon ǀ Chemic-alize.org	ACD/ Labs M1/ M2	COSMO-therm X19 TZVPD-FINE		selected
**Alkyl-amines**	10.21<br>10.7	10.75 / 10.5	10.79 (P10) / 11.2 (P16)	10.64 hexylamine (Perrin 1965)<br>10.7 octylamine (SRC)<br>10.6 octylamine (REACH) (https://echa.europa.eu/registration-dossier/-/registered-dossier/1996/4/22)	10.6
***N*-methyl-alkyl-amines**	10.54	10.78 / 10.9	10.78 (S12)	10.9 N-methylbutylamine (Perrin 1972)	10.8
***N,N*-dimethyl-alkyl-amines**	9.79	9.79 / 10.1	9.63 (T14)	9.99 N,N-dimethylpropylamine<br>10.02 N,N-dimethylbutylamine<br>(H. K. Hall 1957, Correlation of the Base Strengths of Amines 1, JACS, 79 (20) 5441-5444) doi: 10.1021/JA01577A030)	10.0

Table S4: Solvents

Solvent	Purpose	Grade	Supplier
Methanol	Test chemical solution for aquaria, extraction, SPE column, UPLC mobile phase	Lichrosolve	Merck
Milli-Q water	SPE column, dilution of purified extract, UPLC mobile phase		
Ammonium hydroxide	Adjust pH of Milli-Q		ACS
Trifluoroacetic acid	SPE column	HiPerSolve Chroma-norm for LC/MS	VWR Chemicals
Ammonium acetate	UPLC mobile phase	p.a.	Merck

Table S5: Sampling schedule

Day	hours	Water	Fish	TOC	Alkalinity
1	0	X			
1	2	X			
1	4	X			
1	6	X	X		X
1	8	X			
2	24	X	X	X	X
2	28	X			
2	36	X	X		
3	48	X	X	X	
4	72	X		X	
5	96	X		X	
6	120	X		X	
7	144	X	X	X	X
8	168	X		X	
9	192	X		X	
10	216	X	X	X	
11	240	X		X	
12	264	X		X	
13	288	X	X	X	
14	312	X		X	X
15	336	X	X		
Transfer to elimination aquarium					
15	336.5	X			
15	338	X			
15#	340	X			
15	342	X	X	X	
15#	344	X			
16	360	X	X	X	X
17	384	X	X		
18	408	X	X		
21	480	X	X		X
24	552		X		
28	648		X		
32	744	X	X		X
37	864		X		
43	1008	X	X		X

MIX1pH only

Table S6: Mean relative standard deviation (in %) of test substance concentration in triplicate water samples collected during the exposure phase (n = 21 × 3)

MIX1	Mean RSD[a]	MIX1pH	Mean RSD[b]	MIX2	Mean RSD[c]
P9	2	P9	3	T9	3
T10	6	T10	5	Q10	1
P12	2	P12	3	S12	2
T13	2	T13	3	P13	3
Q14	2	Q14	2	T14	3
P16	4	P16	4	S16	5

[a] Outliers: 1 sample for all analytes (sampling error), 1 sample for P12, T13, Q14, P16 (particle artifact)
[b] Outliers: 1 sample for P12, 3 samples for T13 and Q14, 4 samples for P16 (particle artifact)
[c] Outliers: 4 samples for S12, P13, T14, S16 (particle artifact)

Table S7: Test substance concentration range (µg L^{-1}) in blank samples from the water analysis (n = 12 for each mixture)

MIX1	Blank range	MIX1pH	Blank range	MIX2	Blank range
P9	0.04-0.35	P9	0.06-0.28	T9	0.03-0.35
T10	nd[a]	T10	nd-0.1	Q10	0.03-0.33
P12	0.04-0.57	P12	0.03-0.25	S12	0.02-0.05
T13	0.02-0.15	T13	nd-0.03	P13	0.02-0.08
Q14	0.07-0.71	Q14	0.04-0.16	T14	nq[b]
P16	nd-0.17	P16	nd	S16	nq

[a] nd = non-detect
[b] nq = not quantified

Table S8: Mean relative difference (in %) of test substance concentration in duplicate analyses of raw fish extracts

**MIX1** (n = 16)		**MIX2** (n = 3)	
P9	<LOD	T9	3
T10	26[a]	Q10	7
P12	5	S12	2
T13	2	P13	7
Q14	2	T14	2
P16	7	S16	3

[a] One of the duplicate samples was strongly impacted by blanks and the concentrations lay between the LOD and LOQ.

Table S9: Mean relative difference of test substance concentration in duplicate analyses of fish homogenate

**MIX1** (n = 3)		**MIX2** (n = 0)	
P9	<LOD	T9	
T10	16	Q10	
P12	6	S12	
T13	1	P13	
Q14	3	T14	
P16	5	S16	

Table S10: Recovery (mean ± standard deviation in %) of test chemicals spiked to fish homogenate or raw extract

**MIX1**	**Recovery (%)**	**MIX2**	**Recovery (%)**
P9	92±6	T9	103±6
T10	100±3	Q10	95±3
P12	98±2	S12	106±4
T13	97±4	P13	99±4
Q14	92±7	T14	106±2
P16	106±3	S16	116±7

Table S11: Limit of quantification (LOQ, ng g^{-1}) for fish analysis (determined as 10 × standard deviation in procedural blanks and control fish)

**MIX1**	**LOQ**	**MIX2**	**LOQ**
P9	3	T9	4
T10	3	Q10	4
P12	4	S12	7
T13	5	P13	8
Q14	4	T14	6
P16	7	S16	9

Table S12: Alkalinity and pH during the MIX1§ and MIX2 experiments

**Day**	**hour**	**pH**		**Alkalinity (mmol L^{-1})**	
		**MIX1**	**MIX2**	**MIX1**	**MIX2**
Exposure					
1	6	7.58	7.57	1.3137	1.3128
2	24	7.57	7.59	1.2969	1.2905
7	144	7.64	7.59	1.2977	1.2990
14	312	7.66	7.72	1.2663	1.2689
Elimination					
16	360	7.78	7.75	1.2680	1.2705
21	480	7.75	7.75	1.2987	1.3012
32	744	7.81	7.81	1.3017	1.2951
43	1008	7.9	7.92	1.2177	1.2131

§ During the MIX1pH experiment alkalinity was strongly affected by the addition of formic acid.

Table S13: TOC during the MIX1[a] and MIX2 experiments[b]

**Day**	**hours**	**TOC (mg C L^{-1})**	
		**MIX1**	**MIX2**
	Exposure		
2	24	5.7	6.2
3	48	5.8	5.7
4	72	5.4	5.7
5	96	5.5	5.8
6	120	5.3	5.4
7	144	6.1	5.9
8	168	5.4	5.6
9	192	5.2	5.7
10	216	5. 6	5.7
11	240	5.1	5.4
12	264	5.2	5.2
13	288	5.3	5.1
14	312	5.6	5.4
	Elimination		
15	342	5.1	4.7
16	360	5.1	5.2

[a] During the MIX1pH experiment TOC was strongly affected by the addition of formic acid.
[b] There was little variability during the experiment or between experiments. The methanol from the infusion solution contributed 0.8 mg C L^{-1} to the TOC, while the remainder originated from the fish, the fish feed and organic carbon in the raw water supply (~4 mg C L^{-1}), some of which passed through the activated carbon filters in the aquarium water treatment system. In the MIX1pH experiment meaningful measurements of TOC were not possible due to the background signal from the formic acid.

Table S14: Concentrations (μg L^{-1}) of test chemicals in water during the MIX1 experiment. Each data point is the mean of triplicate samples.

Day	hours	P9	T10	P12	T13	Q14	P16
Exposure							
1	0	*118.8*	*21.6*	*29.0*	*7.4*	*22.8*	*1.41*
1	2	*111.1*	*20.1*	*25.0*	*6.7*	*23.2*	*1.37*
1	4	*119.3*	*19.6*	*24.8*	*6.5*	*25.3*	*1.45*
1	6	*110.7*	*18.9*	*23.8*	*6.4*	*24.6*	*1.41*
1	8	*111.9*	*19.6*	*23.2*	*6.5*	*25.1*	*1.43*
2	24	*109.1*	*18.9*	*24.3*	*7.0*	*26.2*	*1.50*
2	28	*115.4*	*19.6*	*24.6*	*7.1*	*26.5*	*1.47*
2	36	*109.1*	*19.3*	*24.2*	*7.0*	*26.6*	*1.49*
3	48	*110.1*	*19.8*	*24.4*	*7.2*	*25.3*	*1.46*
4	72	*107.9*	*19.2*	*24.4*	*7.2*	*25.1*	*1.41*
5	96	*108.4*	*19.0*	*24.4*	*7.4*	*25.8*	*1.49*
6	120	*108.0*	*19.3*	*24.8*	*7.8*	*25.7*	*1.49*
7	144	*105.5*	*19.4*	*24.0*	*7.3*	*25.2*	*1.41*
8	168	*104.8*	*19.3*	*23.8*	*7.7*	*25.6*	*1.52*
9	192	*108.7*	*18.5*	*24.6*	*7.7*	*26.3*	*1.51*
10	216	*102.5*	*18.8*	*23.8*	*7.7*	*25.3*	*1.61*
11	240	*102.8*	*18.0*	*24.2*	*7.8*	*25.4*	*1.63*
12	264	*98.2*	*19.5*	*22.9*	*7.8*	*24.3*	*1.58*
13	288	*96.7*	*17.3*	*21.9*	*7.0*	*23.7*	*1.55*
14	312	*94.9*	*19.4*	*22.8*	*7.5*	*24.5*	*1.73*
15	336	*97.5*	*17.4*	*22.2*	*7.3*	*23.9*	*1.54*
Mean		107	19.2	24.2	7.2	25.1	1.50
RSD (%)		6.3	4.7	5.8	6.2	4.1	5.8
Target		100	25	25	10	25	2.5
% of target		107	77	97	72	100	60
Elimination							
15	336.5	*0.57*	*0.54*	*0.82*	*0.20*	*0.35*	*0.10*
15	338	*0.70*	*0.83*	*0.99*	*0.30*	*0.34*	*0.11*
15	342	*0.46*	*0.81*	*0.79*	*0.28*	*0.27*	*0.08*
16	360	*0.12*	*0.13*	*0.40*	*0.16*	*0.23*	*0.06*
17	384	*0.21*	*0.08*	*0.36*	*0.14*	*0.25*	*0.08*[a]
18	408	*0.13*		*0.36*	*0.14*	*0.32*	*0.07*
21	480	*0.09*		*0.19*	*0.06*	*0.15*	*0.06*
32	744	*0.02*		*0.11*	*0.03*	*0.12*	*0.06*
43	1008	*0.07*		*0.05*	*0.01*	*0.08*	

[a] Concentrations blank corrected, lie between LOD and LOQ

Table S15: Concentrations (µg L^{-1}) of test chemicals in water during the MIX2 experiment. Each data point is the mean of triplicate samples.

Day	hours	T9	Q10	S12	P13	T14	S16
Exposure							
1	0	*52*	*56*	*23.8*	*6.4*	*1.37*	*1.22*
1	2	*49*	*55*	*21.0*	*6.2*	*1.61*	*1.46*
1	4	*49*	*56*	*20.1*	*5.7*	*1.45*	*1.36*
1	6	*50*	*56*	*20.1*	*6.0*	*1.56*	*1.52*
1	8	*51*	*57*	*20.8*	*6.3*	*1.69*	*1.67*
2	24	*49*	*56*	*22.1*	*6.6*	*1.68*	*1.51*
2	28	*54*	*58*	*21.9*	*6.4*	*1.70*	*1.51*
2	36	*53*	*57*	*22.4*	*6.3*	*1.60*	*1.43*
3	48	*51*	*55*	*22.7*	*6.7*	*1.80*	*1.53*
4	72	*49*	*54*	*22.2*	*6.5*	*1.66*	*1.50*
5	96	*50*	*55*	*22.9*	*6.7*	*1.79*	*1.64*
6	120	*51*	*55*	*22.7*	*6.4*	*1.56*	*1.66*
7	144	*50*	*54*	*21.9*	*6.4*	*1.67*	*1.72*
8	168	*52*	*55*	*22.4*	*6.5*	*1.80*	*1.65*
9	192	*51*	*57*	*23.1*	*6.6*	*1.82*	*1.61*
10	216	*50*	*54*	*22.2*	*6.3*	*1.80*	*1.60*
11	240	*50*	*55*	*23.4*	*6.7*	*1.83*	*1.74*
12	264	*49*	*53*	*22.0*	*6.3*	*1.74*	*1.69*
13	288	*47*	*52*	*21.3*	*6.0*	*1.59*	*1.79*
14	312	*48*	*54*	*21.9*	*6.5*	*1.65*	*1.89*
15	336	*51*	*55*	*22.7*	*6.9*	*1.84*	*1.85*
Mean		50	55	22.1	6.4	1.68	1.60
RSD (%)		3.1	2.3	4.4	4.1	7.5	10.2
Target		50	50	25	10	2.5	2.5
% of target		100	110	88	64	68	64
Elimination							
15	336.5	*0.57*	*0.06*	*0.55*	*0.20*	*0.04*	*0.06*
15	338	*0.89*	*0.03*	*0.96*	*0.22*	*0.05*	*0.08*
15	342	*0.76*	*0.05*	*1.03*	*0.16*	*0.04*	*0.05*
16	360	*0.06*	*0.05*	*0.53*	*0.11*	*0.03*	*0.04*
17	384	*0.03*	*0.08*	*0.36*	*0.09*	*0.03*	*0.04*
18	408	*0.03*	*0.04*	*0.28*	*0.09*	*0.03*	*0.04*
21	480	*0.02*	*0.05*	*0.11*	*0.06*	*0.02*	*0.03*
32	744	*0.03*	*0.05*	*0.04*	*0.05*	*0.02*	*0.04*
43	1008		*0.02*	*0.02*	*0.04*		

Table S16: Concentrations (µg L^{-1}) of test chemicals in water during the MIX1pH experiment. Each data point is the mean of triplicate samples.

Day	hours	P9	T10	P12	T13	Q14	P16
Exposure							
1	0	119	23.6	30.4	7.1	22.9	1.37
1	2	116	22.0	28.4	6.9	23.9	1.38
1	4	108	20.8	26.8	6.9	24.4	1.38
1	6	121	24.7	31.5	8.3	28.2	1.63
1	8	115	23.9	30.1	8.0	26.5	1.66
2	24	112	22.8	29.6	7.9	26.6	1.54
2	36	118	24.2	30.9	8.1	25.8	1.56
3	48	116	23.0	30.3	8.3	27.3	1.68
4	72	108	22.1	28.5	8.2	27.2	1.59
5	96	110	22.2	29.4	8.0	26.4	1.71
6	120	107	22.0	28.2	7.8	26.1	1.58
7	144	107	21.2	27.7	7.9	26.1	1.64
8	168	112	22.5	29.6	8.4	27.5	1.68
9	192	114	23.3	29.1	8.2	26.5	1.70
10	216	111	21.4	27.9	7.7	25.0	1.46
11	240	109	21.2	28.6	7.9	26.0	1.51
12	264	115	22.1	29.1	7.8	26.2	1.60
13	288	108	21.0	26.3	7.2	24.1	1.37
14	312	115	21.9	28.5	8.0	25.6	1.44
15	336	112	21.9	28.8	8.1	25.3	1.43
Mean		113	22.4	29.0	7.8	25.9	1.55
RSD (%)		3.7	4.9	4.5	5.8	5.1	7.7
Target		100	25	25	10	25	2.5
% of target		113	90	116	78	104	62
Elimination[a]							
15	336.5	0.28	0.37	0.32	0.16	0.31	0.05
15	338	0.25	0.51	0.54	0.34	0.28	0.09
15	340	0.28	0.54	0.59	0.38	0.33	0.10
15	342	0.17	0.49	0.46	0.34	0.21	0.13
15	344	0.20	0.52	0.50	0.33	0.33	0.13
16	360	0.17	0.27	0.23	0.14	0.21	0.08
17	384	0.17	0.19	0.17	0.08	0.20	0.06[b]
18	408	0.07	0.16	0.07	0.05	0.12	0.05
21	480	0.07	0.12	0.06	0.03	0.10	0.04
28	648	0.07	0.07	0.06	0.02	0.18	0.03
32	744	0.13	0.08	0.07	0.03	0.12	0.07
43	1008	0.09		0.06	0.01	0.09	

[a] During the MIX1pH experiment a higher sampling frequency was employed at the beginning of the elimination phase, and a pulse in the concentration of T10, P12, and T13 in water was observed during the first hours after adding the fish to the elimination aquarium. This was presumably caused by depuration of the test chemicals by the fish. However, within 2 days the concentrations of all chemicals had fallen to a level at least a factor of 50 below the concentrations in the exposure phase. The one exception was P16, for which the decrease was a factor of ~20.

[b] Concentrations blank corrected, lie between LOD and LOQ

Table S17: Concentrations (ng g^{-1} ww) of test chemicals in fish during the exposure phase of the MIX1 experiment.

Day	hours	P9	T10	P12	T13	Q14	P16
1	6	46	1005	156	1276	57	67
1	6	369	1035	2949	1400	49	270
1	6	195	1204	2736	1684	47	292
2	24	254	2026	5200	3828	75	793
2	24	55	1827	751	4003	92	432
2	24	349	1983	6604	4074	89	968
2	36	355	1748	7711	4936	95	1371
2	36	292	2351	7715	5990	123	1412
3	48	57	1745	763	6741	149	843
3	48	150	2149	5457	5935	124	1538
3	48	77	2102	1166	7114	174	799
7	144	487	2041	10816	11202	319	3525
7	144	235	2492	5563	12950	334	2947
7	144	165	2535	3782	13222	309	3101
10	216	693	2751	14523	14667	455	4999
10	216	856	3473	19268	17276	580	6184
10	216	693	3211	14716	14121	501	4557
13	288	547	2826	13992	18432	689	5951
13	288	154	2919	3812	16588	635	3296
13	288	976	3529	19113	20280	554	7050
15	336	115	2762	1598	19788	734	2026
15	336	129	2951	2448	21365	563	3217
15	336	405	3258	9703	16864	581	5441

Table S18: Concentrations (ng g^{-1} ww) of test chemicals in fish during the elimination phase of the MIX1 experiment.

Day	hours	P9	T10	P12	T13	Q14	P16
15	342	299	1359	16257	15495	766	5769
15	342	25	1608	1371	20065	547	3474
15	342	11.7	1227	2571	13970	610	4530
16	360	8.4	586	2022	19163	528	5433
16	360	59	445	13516	17776	515	7372
16	360	5.1	256	3296	14430	639	5047
17	384		121	988	13423	525	3936
17	384		88	1944	12902	576	4225
17	384		92	10328	13740	510	5785
18	408		36	1314	11513	621	4346
18	408		83	4967	16628	590	5435
18	408		53	3015	12977	588	3698
21	480		12	1614	8334	446	3112
21	480		12	451	6680	564	3040
21	480		12	295	10632	445	2566
24	552		10	175	6960	471	1943
24	552		5	688	7893	347	3572
24	552		10	3968	4552	385	4369
28	648			1648	4095	326	3692
28	648			261	3077	309	2667
28	648			2010	5341	389	2867
32	744			123	2211	231	1831
32	744			132	2563	281	1801
32	744			261	1581	249	1547
37	864			66	1789	290	1140
37	864			70	1887	275	1755
43	1008			321	802	160	1897
43	1008			20	888	199	1449
43	1008			141	1171	264	1429

Table S19: Concentrations (ng g^{-1} ww) of test chemicals in fish during the exposure phase of the MIX2 experiment.

Day	hours	T9	Q10	S12	P13	T14	S16
1	6	714	5.9	2044	155	316	224
1	6	801	0.6	2504	430	223	234
1	6	813	1.8	1851	80	342	232
2	24	1101	2.1	4312	439	947	814
2	24	1289	4.6	5804	856	1207	946
2	24	1143	6.1	5488	744	972	1072
2	36	1400	4.1	6907	1054	1508	1383
2	36	1296	3.6	5723	484	1452	1250
3	48	1749	6.1	8412	2242	2149	2352
3	48	1108	4.5	5529	722	1951	1723
3	48	1411	3.7	7647	968	2007	1683
7	144	1576	5.4	11933	2695	5383	4415
7	144	1408	10.8	11110	3511	4807	4334
7	144	1526	6.7	11889	2666	5108	5117
10	216	2284	11.9	17361	13542	8085	6821
10	216	1616	7.6	14240	11565	6461	5672
10	216	2214	13.2	16321	4310	7885	6241
13	288	2180	10.9	18782	4229	9510	13655
13	288	2281	11.9	16935	4176	8002	7286
13	288	3026	12.4	20005	14531	8874	7356
15	336	2042	8.7	16795	2672	10008	7785
15	336	1933	11.4	15143	1861	7934	7156
15	336	2292	13	17482	6121	8980	9853

Concentrations blank corrected, lie between LOD and LOQ

Table S20: Concentrations (ng g^{-1} ww) of test chemicals in fish during the elimination phase of the MIX2 experiment.

Day	hours	T9	Q10	S12	P13	T14	S16
15	*342*	741	12	16392	6685	10305	8035
15	*342*	920	8	12294	2829	9311	7043
15	*342*	847	7	20378	15761	10236	8107
16	*360*	519	9	10773	6949	6790	6367
16	*360*	174	3.9	12920	5087	7392	9085
16	*360*	660	12	11666	5334	7294	6500
17	*384*	73		9592	10407	8218	6091
17	*384*	133		9481	4936	6771	6012
17	*384*	52		9797	10347	6472	5634
18	*408*	17.5		10492	12394	8870	6030
18	*408*	33.4		8083	3833	7510	5748
18	*408*	46.8		9560	10057	7958	5763
21	*480*			4065	1198	5735	4548
21	*480*			4742	5609	5121	4560
21	*480*			4901	2001	6208	5414
24	*552*			2728	3550	4533	4489
24	*552*			3468	2144	4126	5430
24	*552*			2401	1319	5100	4676
28	*648*			1471	2224	3780	3059
28	*648*			1951	2850	3329	3171
28	*648*			2022	1249	3164	3912
32	*744*			1808	2377	4233	3350
32	*744*			1434	2481	2656	2933
32	*744*			1287	1884	1711	2522
37	*864*			765	421	2057	2261
37	*864*			986	1350	1639	2441
37	*864*			616	1274	2017	2829
43	*1008*			675	1138	1680	2163
43	*1008*			164	136	783	1547
43	*1008*			215	709	955	1738

Concentrations blank corrected, lie between LOD and LOQ

Table S21: Concentrations (ng g^{-1} ww) of test chemicals in fish during the exposure phase of the MIX1pH experiment.

Day	hours	P9§	T10	P12	T13	Q14	P16
1	6	32.0	69	106	445	58	169
1	6	29.3	96	52	502	47	109
1	6	34.7	80	399	435	45	260
2	24	34.5	149	108	1048	99	394
2	24	31.2	116	92	848	141	377
2	24	32.6	139	103	1058	135	362
2	36	31.4	146	149	1196	192	593
2	36	28.6	145	131	1358	155	504
3	36	31.5	168	151	1271	156	510
3	48	23.7	185	1158&	1795	181	1688
3	48	4.0	132	337	1512	204	1242
3	48	31.1	135	168	1390	248	904
7	144	34.4	203	205	2199	398	1769
7	144	38.9	177	133	2235	416	1245
7	144	37.9	236	156	2488	358	1586
10	216	34.8	267	142	2702	689	1823
10	216	30.0	176	176	2846	360	1755
10	216	31.5	156	170	2274	500	1531
13	288	49	238	1904&	3100	475	5611
13	288	52	259	1829&	3218	541	5426
13	288	46	209	181	2779	739	1873
15	336	44	208	206	2828	813	2374
15	336	31	62	187	2632	846	2061
15	336	44.2	186	475	2557	571	3284

§ Concentrations not blank corrected, blank 34±5 ng g^{-1} ww
# Concentrations blank corrected, lie between LOD and LOQ
& Treated as an outlier in the model fitting

Table S22: Concentrations (ng g^{-1} ww) of test chemicals in fish during the elimination phase of the MIX1pH experiment.

Day	hours	P9	T10	P12	T13	Q14	P16
15	338		128	168	2567	852	2142
15	338	6.7	126	275	2821	719	2968
15	338	4.1	128	95	3012	1367	2240
15	340	3.5	59	180	1767	801	2183
15	340	3.8	143	380	3251	817	4320
15	340	3.9	71	298	2549	466	3627
15	344	4.0	83	279	2525	703	3690
15	344	3.1	49	206	2010	744	2703
15	344		75	109	2446	644	2564
16	360		9.9	64	1625	383	1874
16	360		14.7	26.6	1728	595	1287
16	360		11.9	46	1555	794	2102
17	384		6.3	31.2	685	635	2094
17	384		8.7	28.1	1261	594	1270
17	384		8.7	9.1	837	731	2436
18	408		4.9	11.4	401	677	1453
18	408		5.8	70	584	760	3667
18	408		6.3	48	578	490	4044
21	480			16.9	311	382	2194
21	480		4.5	6.8	406	532	2544
21	480		4.0	18.7	290	416	3621
24	552				181	379	1331
24	552				153	413	1634
24	552				133	449	3011
28	648				42	449	863
28	648				50	508	1802
28	648				70	384	1677
32	744				30.3	309	1389
32	744				37.7	371	1464
32	744				47	500	1495
37	864				13.1	203	621
37	864				30.9	306	926
37	864				42	315	300
38	888				289	270	879
38	888				18.8	224	459
38	888				20.0	193	464

Table S23: Inter-individual variability of test chemical concentrations in fish, expressed as the relative standard deviation in % for the 3 fish sampled at each time point (mean across all time points ± standard deviation).

MIX1	RSD	MIX1pH	RSD	MIX2	RSD
P9	54±27	P9		T9	12±7
T10	11±4	T10	20±14	Q10	34±30
P12	61±37	P12	47±40	S12	12±5
T13	10±3	T13	9±3	P13	55±22
Q14	12±4	Q14	18±7	T14	10±6
P16	31±20	P16	23±16	S16	15±11

Table S24: Course approximation of relative concentrations (with respect to the parent substance) of demethylation products of test chemicals found in 3 fish sampled at the end of the exposure period, assuming that response factors of parent and product were similar. The relative concentration is given in brackets below the identity of the product.

Parent	T9<br>(1)	T10<br>(1)	S12<br>(1)	T13<br>(1)	T14<br>(1)	S16<br>(1)
Product 1	S9<br>(0.2)	S10<br>(0.15)	P12<br>(0.005)	S13<br>(0.05)	S14<br>(0.1)	P16<br>(nd)
Product 2	P9<br>(0.02)	P10<br>(nd)		P13<br>(0.001)	P14<br>(nd)	

Table S25: Comparison of k_U and k_U/D_{MLW} for Q14, S16 and P16

Chemical	Uptake rate constant (k_U, L kg^{-1} h^{-1})		k_U/D_{MLW}	
	pH 7.6	pH 6.2	pH 7.6	pH 6.2
Q14	0.104	0.121	3.6E-07	4.2E-07
S16	22		4.3E-07	
P16	15.3	7.8	1.9E-07	9.8E-08

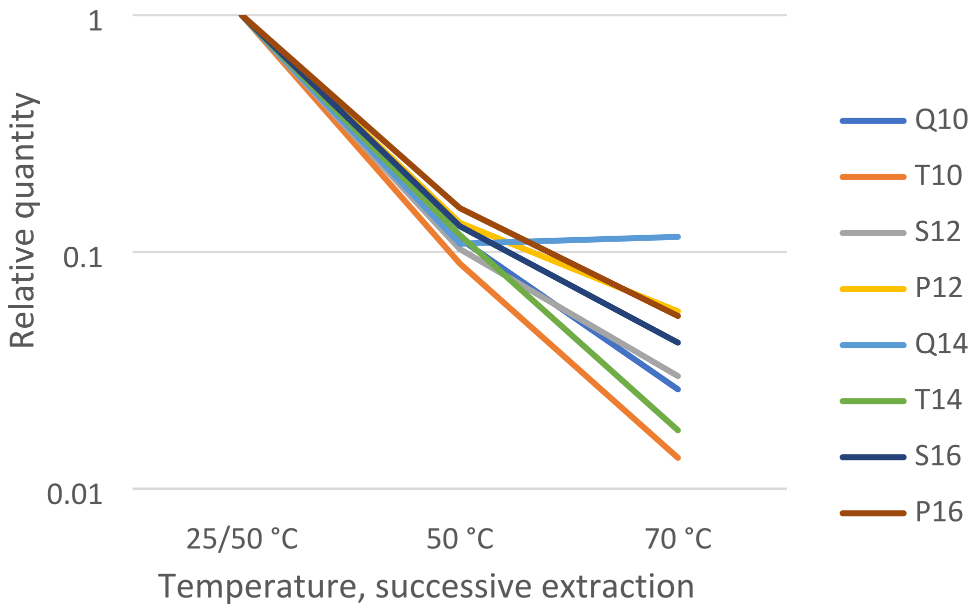

Figure S1: Relative quantity extracted from fish homogenate in successive batch extractions at different temperatures. 500 mg of homogenate was extracted in 8 mL of methanol after addition of internal standard. The slurry was sonicated for 1 h followed by centrifugation, decanting of the extract, and re-extraction of the residue in fresh solvent and internal standard. The first extraction was conducted at 25 °C for 0.5 h followed by 50 °C for 0.5 h.

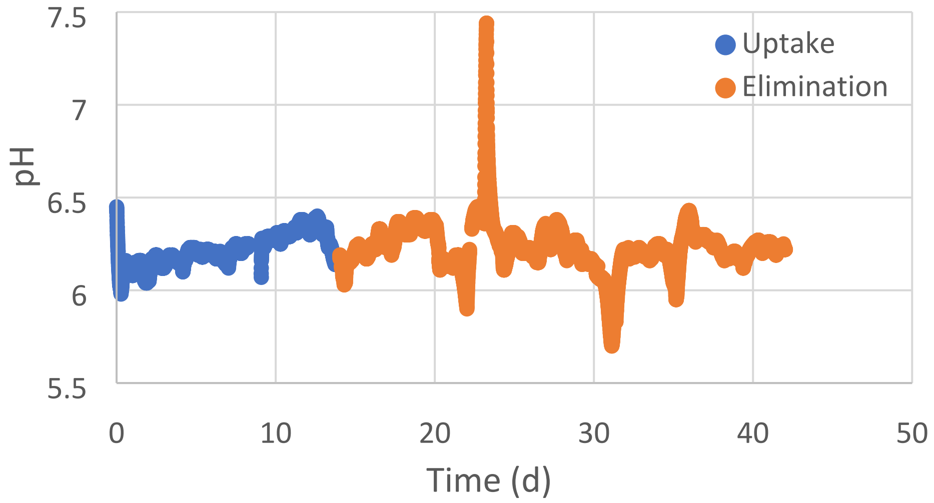

Figure S2: pH during the MIX1pH experiment. There positive spike during the elimination phase was due to a defect in the formic acid supply system. The period of elevated pH was ~6 h.

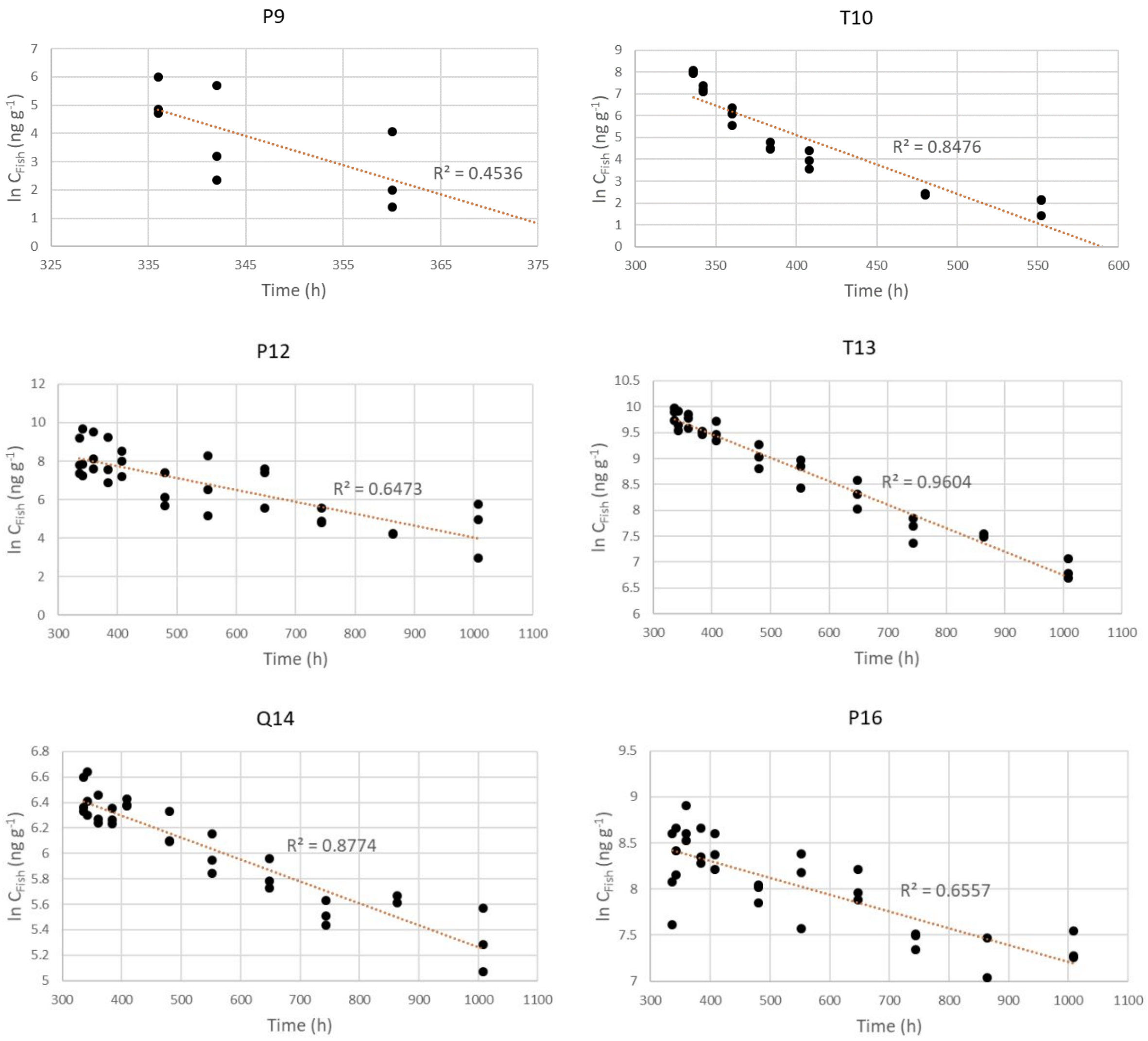

Figure S3: Semilogarithmic plot of chemical elimination during the MIX1 experiment.

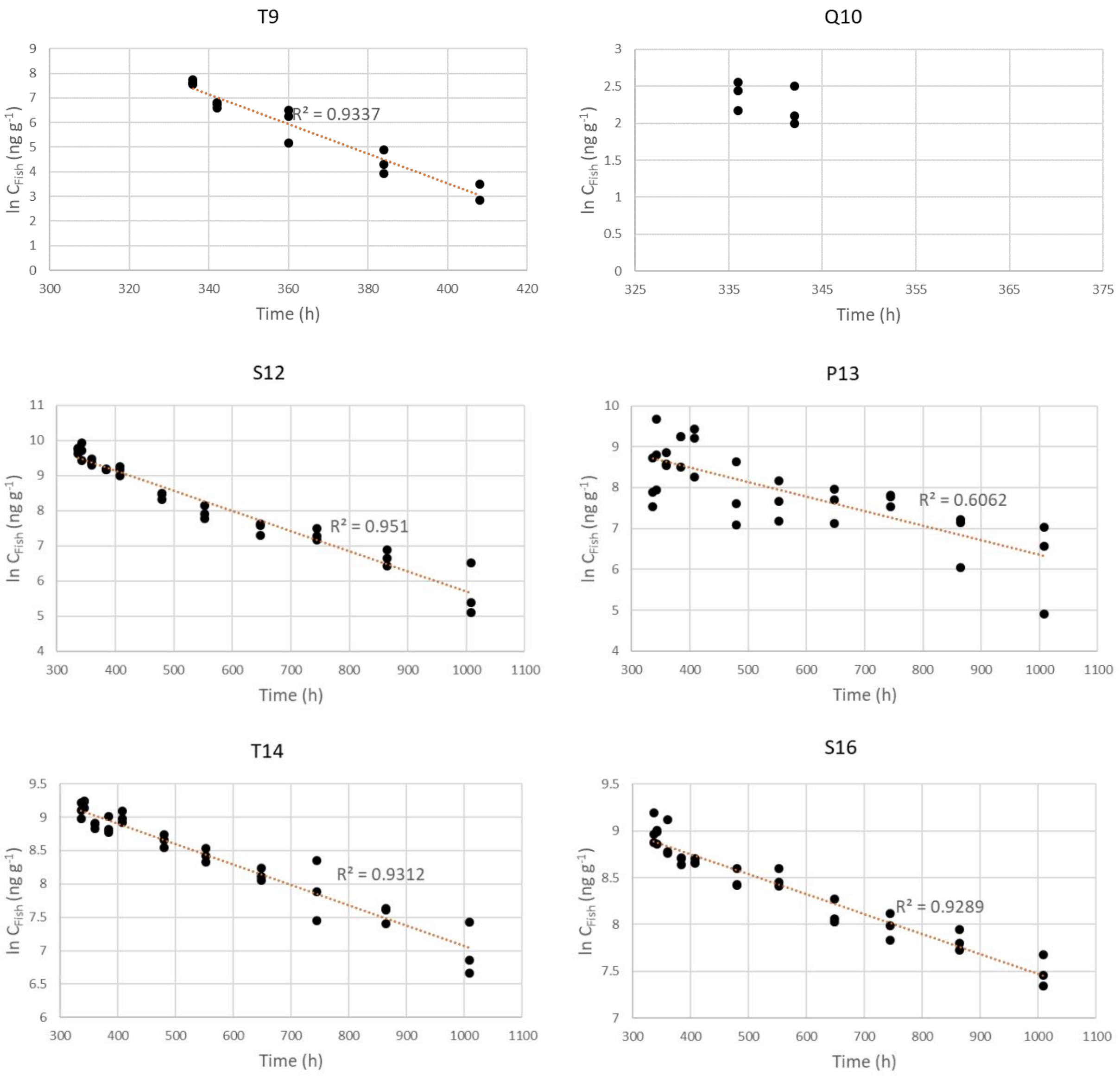

Figure S4: Semilogarithmic plot of chemical elimination during the MIX2 experiment.

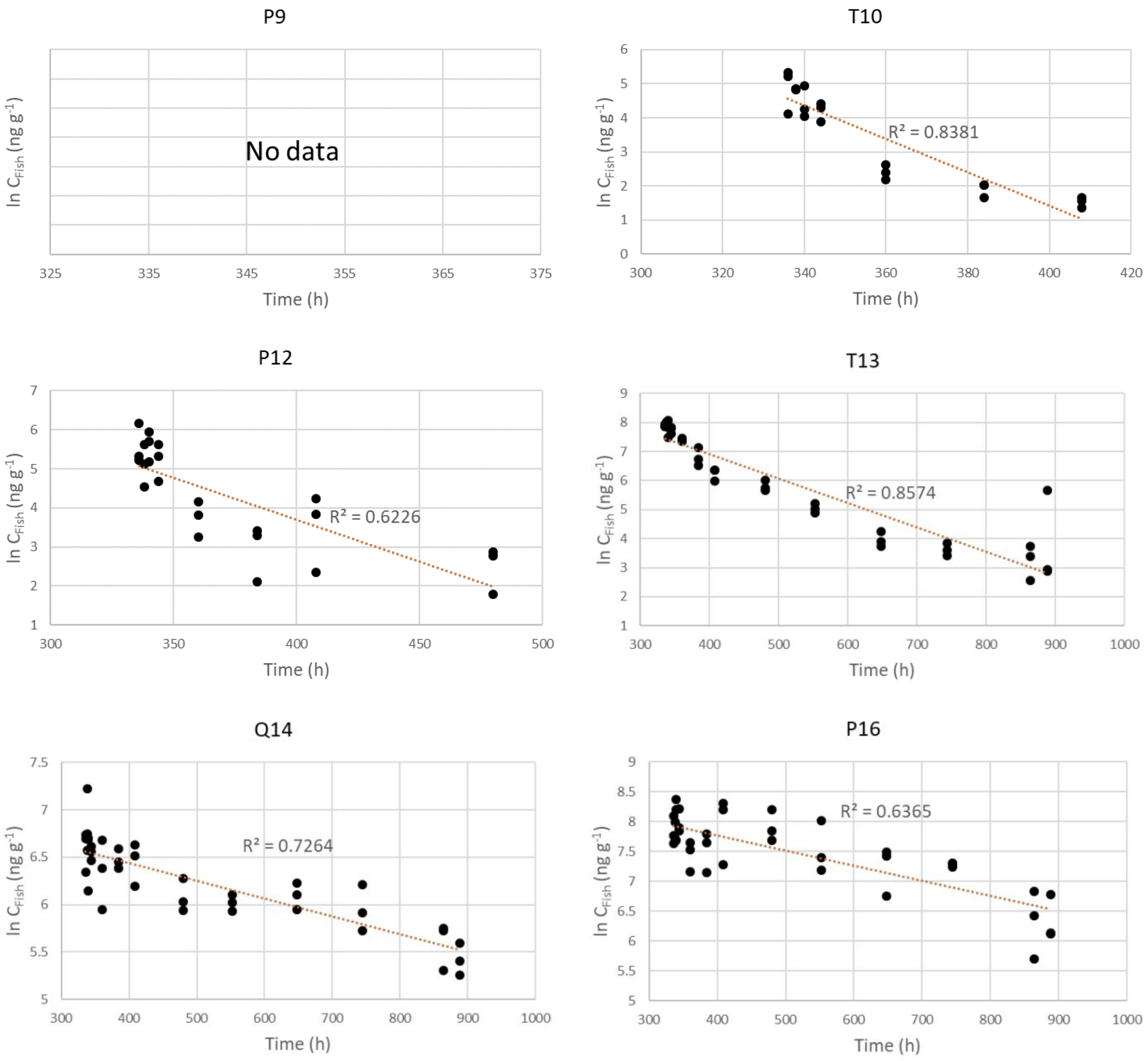

Figure S5: Semilogarithmic plot of chemical elimination during the MIX1pH experiment.

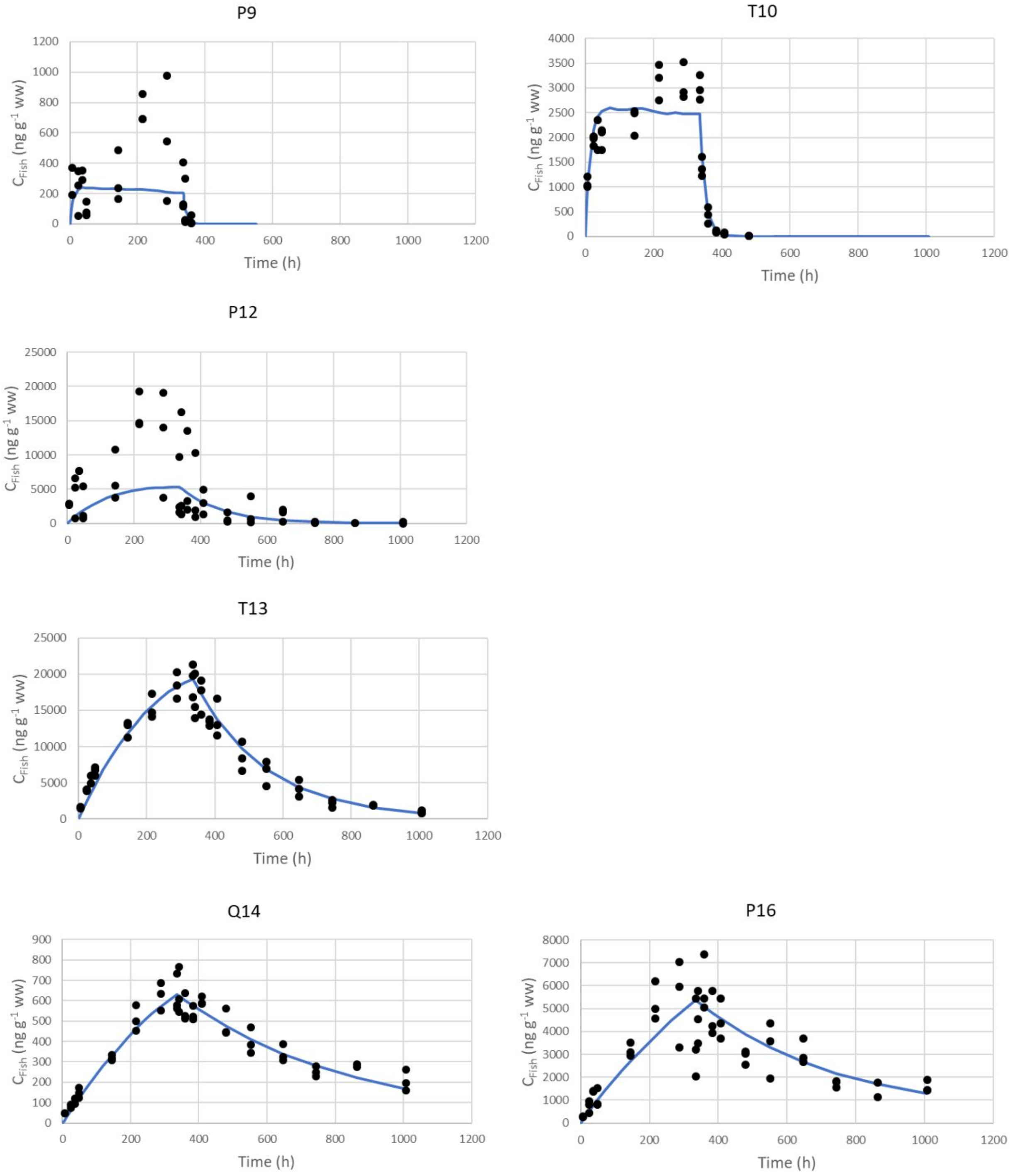

Figure S6: Plot of chemical uptake and elimination during the MIX1 (pH 7.6) experiment showing the model fit to the data.

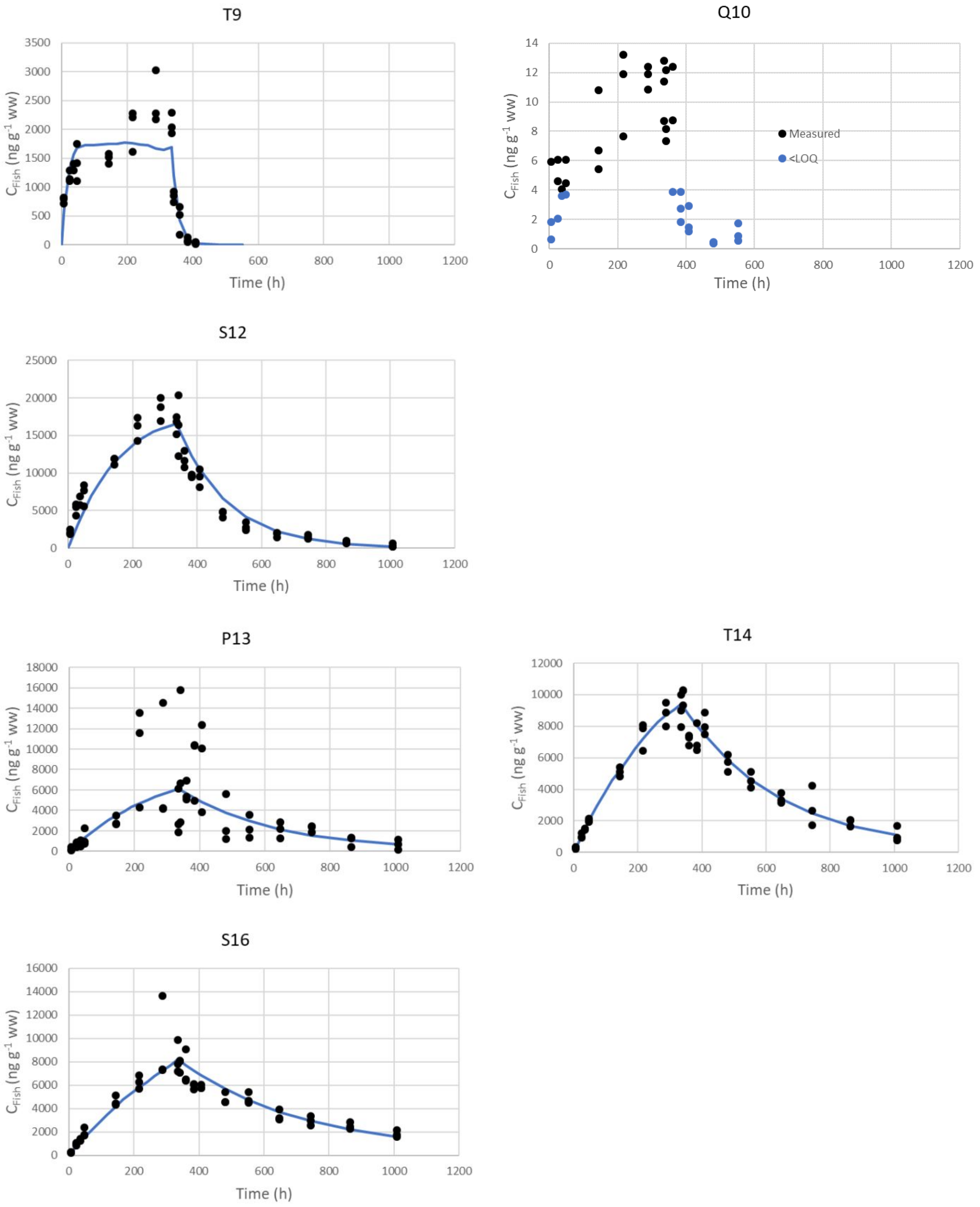

Figure S7: Plot of chemical uptake and elimination during the MIX2 (pH 7.6) experiment showing the model fit to the data.

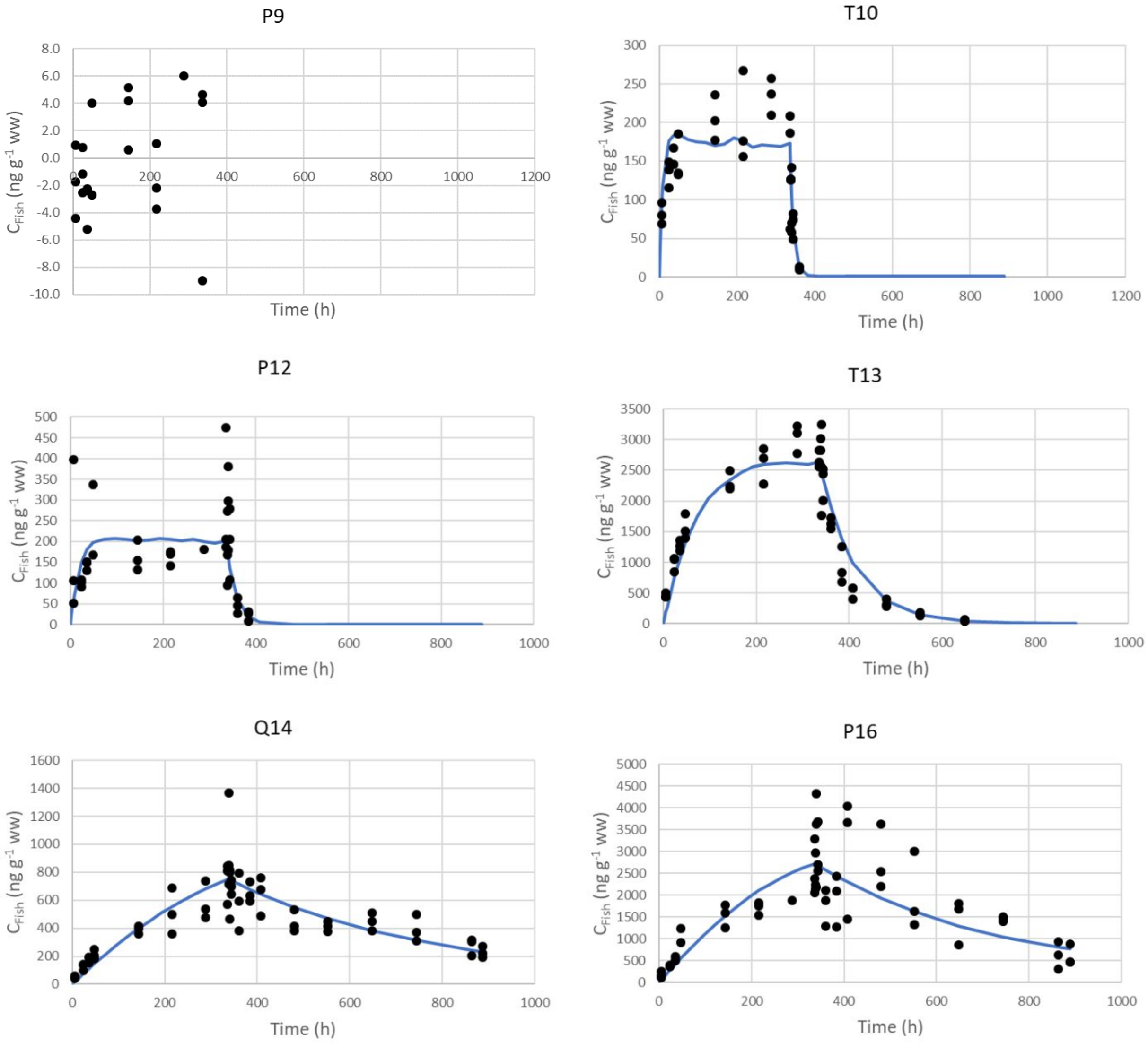

Figure S8: Plot of chemical uptake and elimination during the MIX1pH (pH 6.2) experiment showing the model fit to the data. All data for P9 were below the LOQ.
